# Transcriptome analysis of the prefrontal cortex identifies inflammatory genes associated with cognitive impairment in a model of multiple sclerosis

**DOI:** 10.1038/s41420-026-03051-9

**Published:** 2026-03-25

**Authors:** Luca Zupo, Annalisa Adinolfi, Marco Pieraccioli, Marika Guerra, Valentina Corvino, Francesco Ria, Veronica Ceci, Valerio Chiurchiù, Lorenzo Gaetani, Silvia Sperandei, Massimiliano Di Filippo, Gabriele Di Sante, Maria Concetta Geloso, Claudio Sette

**Affiliations:** 1 Section of Human Anatomy, Department of Neuroscience, Università Cattolica del Sacro Cuore, Rome, Italy; 2 Gemelli Science and Technology Park (GSTeP)-Organoids Research Core Facility, Fondazione Policlinico Agostino Gemelli IRCCS, Rome, Italy; 3 Section of General Pathology, Department of Translational Medicine and Surgery, Università Cattolica del Sacro Cuore, Rome, Italy; 4 Institute of Translational Pharmacology, National Research Council, Rome, Italy; 5 Laboratory of Resolution of Neuroinflammation, IRCCS Fondazione Santa Lucia, Rome, Italy; 6 Section of Neurology, Department of Medicine and Surgery, University of Perugia, Perugia, Italy; 7 Department of Medicine and Surgery, Section of Human, Clinical and Forensic Anatomy, University of Perugia, Perugia, Italy

**Keywords:** Neuroimmunology, Multiple sclerosis

## Abstract

Cognitive impairment (CI) is a hallmark of multiple sclerosis (MS). Despite its relevance, however, knowledge of the key steps involved in its pathogenesis remains incomplete. Consequently, predictive biomarkers and actionable therapeutic options to counteract CI in MS patients are not available. To identify changes associated with CI in MS, we performed transcriptomic analyses of the prefrontal cortex (PFC), a cortical region relevant for cognition, in the experimental autoimmune encephalomyelitis (EAE) mouse model. Our analyses highlighted the strong upregulation of inflammatory pathways in the PFC of EAE mice. Clustering of the top differentially expressed genes (DEGs) in the PFC identified a low (EAE-L) and a high (EAE-H) inflammation subgroup. Notably, enhanced inflammation in the EAE PFC caused increased changes in expression levels of MS-associated genes with relevance for CI. Cell Type-Specific Expression Analysis (CSEA) and morphological analyses indicated that, while EAE-L mice showed only microglia activation, EAEH mice also displayed the involvement of astrocytes, consistent with a more advanced stage of disease. Moreover, neuronal genes were only downregulated in the EAE-H PFC. Analysis of cognitive performance in pre-symptomatic EAE mice revealed that high expression of genes associated with the antigen presentation and the complement pathways was associated with CI. Moreover, expression of C1q complement proteins was increased in the cerebrospinal fluid of MS patients affected by CI. These findings indicate that inflammation in the PFC during EAE is associated with CI and identify a subset of inflammatory genes that may represent early markers and risk factors for functional PFC impairment and loss of cognitive performance in MS patients.

## Introduction

Cognitive impairment (CI) is among the most disabling symptoms of multiple sclerosis (MS). It occurs in approximately two-thirds of MS patients and severely hampers their daily life and employment conditions, independently of sensory-motor deficits [1, 2]. CI involves several domains, such as learning, memory, information processing speed, and executive functions [3], and it is characterized by a progressive course without remissions [4]. The occurrence of neuroaxonal pathology, independent from demyelination, is thought to provide a relevant contribution to CI [5, 6]. In this regard, mounting evidence suggests that neuroinflammation induces marked changes in neuroplasticity and synaptic structure [7], which represent early manifestations of neuronal involvement in MS [5, 7]. For instance, the expression of synaptic proteins that are important for cognitive functions, such as CASK, Neurexins, and their post-synaptic ligands Neuroligins, is altered in the hippocampus and prefrontal cortex (PFC) of MS patients and of the experimental autoimmune encephalomyelitis (EAE) mouse model of MS [8, 9], suggesting that inflammation impacts on the transcriptional signature of these cognitive-associated brain regions. Accordingly, widespread neuron-specific changes in gene expression were detected in the cortical areas affected by neurodegeneration in MS [10]. For instance, excitatory neurons of the upper cortical layers showed a selective vulnerability to meningeal inflammation, associated with concomitant upregulation of stress pathway genes [10]. Furthermore, markers of excitatory neurons were downregulated in nonlesioned cortical areas of MS brains [11], suggesting that even low levels of local inflammation may trigger the onset of neurodegeneration.

Regional impairment of grey matter structures involved in cognitive functions has a direct impact on neuropsychological performance [12, 13]. For instance, regional atrophy affecting the hippocampus [14] or the PFC [12] is associated with memory impairment and executive dysfunction, respectively. Notably, the PFC is involved in MS [15, 16], and frontal/prefrontal lesions are associated with impaired cognitive performances in MS patients [12, 16–18]. CI also occurs in EAE mice [18] and, like in humans, it is associated with dysfunction of cortical regions involved in cognitive functions, including the PFC [19, 20].

The EAE SJL/J mouse model of relapsing-remitting (RR) MS is characterized by lesions extending to rostral areas of the brain and is particularly suited for investigating CI associated with cortical lesions [21, 22]. We previously reported that inflammatory changes in the PFC of EAE SJL/J mice are correlated with signs of CI already at the presymptomatic phase of the disease [9]. High inflammation levels impacted on PFC circuitry and synaptic arrangement [9], in line with the reported association between neuroinflammation and CI in MS [7] and other neurodegenerative diseases [23]. Altered expression of specific inflammatory mediators, such as IL-1β, was associated with memory impairment and executive dysfunction [24]. Likewise, overexpression of soluble mediators of inflammation was shown to play a causative role in the impairment of synaptic plasticity and in the dysfunction of the neuronal networks underlying CI in MS and EAE [25]. Nevertheless, the neurobiology of CI during MS is not fully understood yet. In this study, we carried out a transcriptomic analysis of the mouse PFC at the acute phase of EAE to elucidate the changes in gene expression associated with the disease in this brain region deeply involved in cognition. Our results support the hypothesis that increased levels of neuroinflammation in the PFC play a key role in the pathological changes that underlie cognitive deficits and identify a subset of genes that may represent valuable markers and/or potential targets for MS-associated CI.

## Results

### EAE triggers widespread induction of immune-related and inflammatory genes in the PFC

**Fig. 1:**
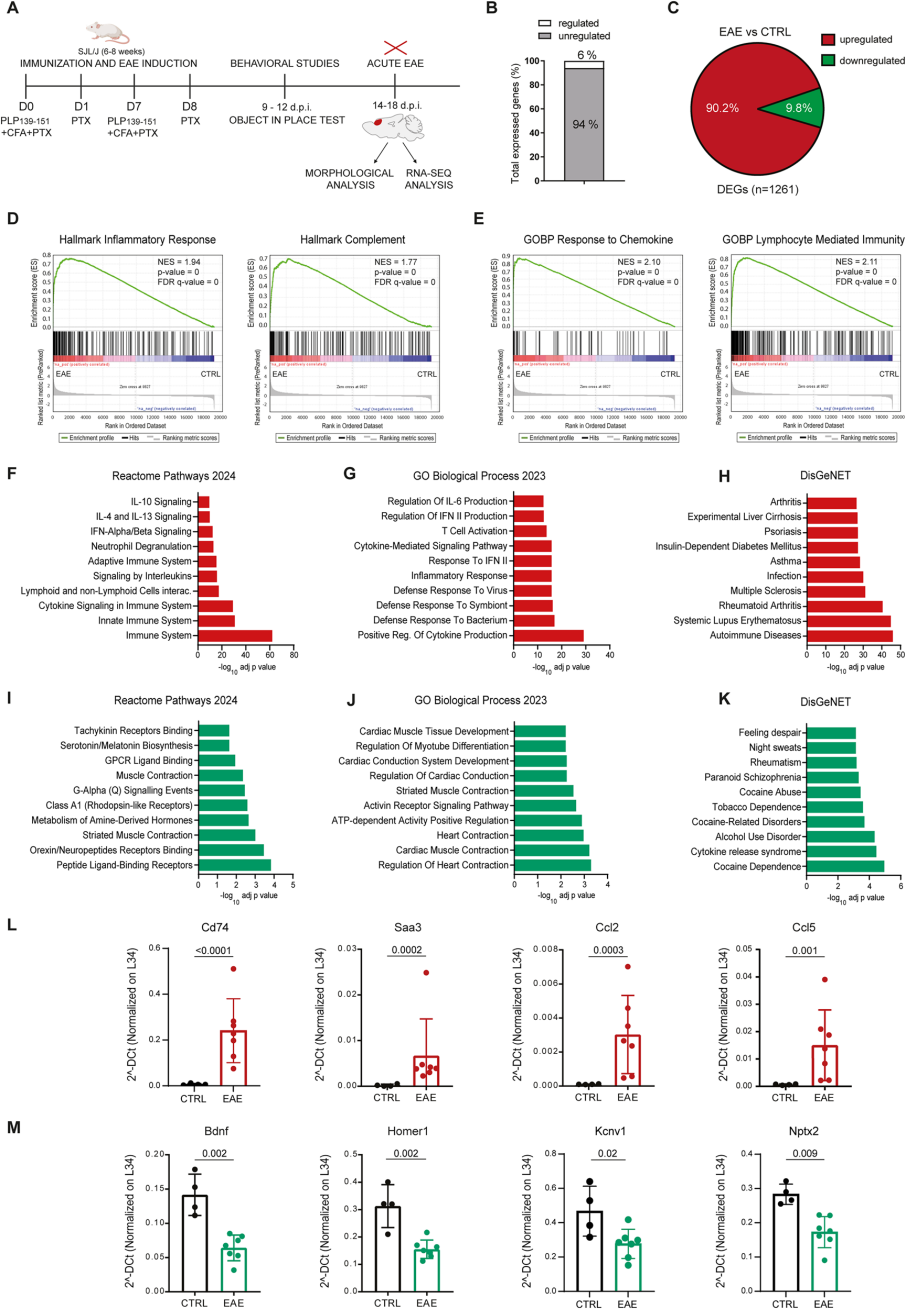
EAE induces widespread reprogramming of the PFC transcriptome. **A** Schematic representation of the experimental design. **B** Graph showing the percentage of regulated and unregulated genes in the PFC of EAE mice compared with controls (CTRL)s. (**C**) Pie chart showing the percentages of upregulated (red) and downregulated (green) genes in the PFC of EAE mice. **D**, **E** Gene Set Enrichment Analysis (GSEA) of the samples of PFC EAE mice vs CTRL. Annotations of upregulated (**F**–**H**) and downregulated (**I**–**K**) genes in the PFC of EAE mice, respectively in Reactome Pathways 2024 (**F**, **I**), Biological Process 2023 (**G**, **J**), DisGeNET (**H**, **K**) databases. **L, M** Bar graphs showing qPCR analyses of the expression of selected upregulated (**L**) and downregulated (**M**) genes in the PFC of CTRL and EAE mice. Student’s *t* Test; *p* < 0.05. All values are given as means ± SD.

Inflammation alters the transcriptional signature of brain regions involved in cognitive functions, including the PFC, thus contributing to the impairment of synaptic structure and function [7, 26–28]. Nevertheless, how MS-associated inflammation modulates the global PFC transcriptome is unknown. To address this issue, we performed RNA-seq analysis of PFC samples isolated from female SJL/J mice immunized with PLP_139-151_ (Fig. 1A). Starting at 13 ± 2 days post-immunization (d.p.i.), all EAE mice (*n* = 7) showed typical clinical symptoms of the disease, reaching the peak of the acute phase at 19 ± 2 d.p.i. (Additional file 1A). The brain of EAE mice showed the presence of typical neuropathological changes, such as subpial and perivascular infiltrates of lymphocytes (CD3^+^ cells) in white and grey matter structures, but no signs of overt neurodegeneration (Additional file 1B, C). Bioinformatic analysis of the RNA-seq dataset revealed a strong impact of EAE on the PFC transcriptome, with significant changes in 6% of all expressed genes (Fig. 1B). The vast majority (90.2%) of these differentially expressed genes (DEGs; fold change ≥1.5, p-adj value < 0.05) were upregulated compared to the control (*n* = 4) PFC samples (Fig. 1c, Additional Table 2).

Gene Ontology (GO) analysis of DEGs highlighted mainly terms related to immune response and inflammation (Additional file 2A, B), which are biological processes directly involved in MS pathogenesis [29]. Coherently, Gene Set Enrichment Analysis (GSEA) revealed a highly significant enrichment in the “inflammatory response” hallmark and the “lymphocyte mediated immunity” and “response to chemokine” biological processes in the EAE PFC (Fig. 1D, E). We also found a significant enrichment in genes associated with the complement system (Fig. 1D), a component of the innate immune response that plays a key role in neurodegeneration [30]. Notably, genes related to these terms were prevalently upregulated by EAE (Fig. 1F, G). Furthermore, query of the DisGeNET database, comprising a collection of genes and variants associated with human diseases[31], revealed that upregulated genes were highly related to autoimmune diseases, including MS (Fig. 1H). By contrast, downregulated genes are related to the neuronal compartment, such as “neuropeptide metabolism and signalling” and “cardiac muscle function” (Fig. 1I, J), a GO term including genes that are also expressed in the CNS and are associated with neuronal functions (i.e. *Bdnf*, *Apnl, Homer1*). This group also includes endothelial genes involved in neurovascular coupling, the process that associates neuronal activity with changes in blood flow, whose altered expression may impact on neuronal functions [32]. Downregulated genes were also associated with neuropsychiatric syndromes, such as cocaine dependency or paranoid schizophrenia (Fig. 1K). Analysis by qPCR on a subset of DEGs confirmed the induction of genes encoding inflammatory factors relevant for the development and progression of both MS and EAE, like *Cd74*, *Saa3*, *Tnf*, *Ccl2* and its receptor *Ccr2*, *Cxcl10* and *Ccl5* [29, 33–35] (Fig. 1L; Additional file 2C). Furthermore, we also confirmed the downregulation of genes involved in neuronal functions, like *Bdnf* and *Homer1* (Fig. 1M). These findings uncover the widespread transcriptome changes occurring in the PFC during the inflammatory and immune-mediated processes that characterize EAE and indicate that this cognitive-relevant brain area is strongly impacted by EAE-associated neuroinflammation.

### Identification of two EAE-associated transcriptional states in the PFC

**Fig. 2:**
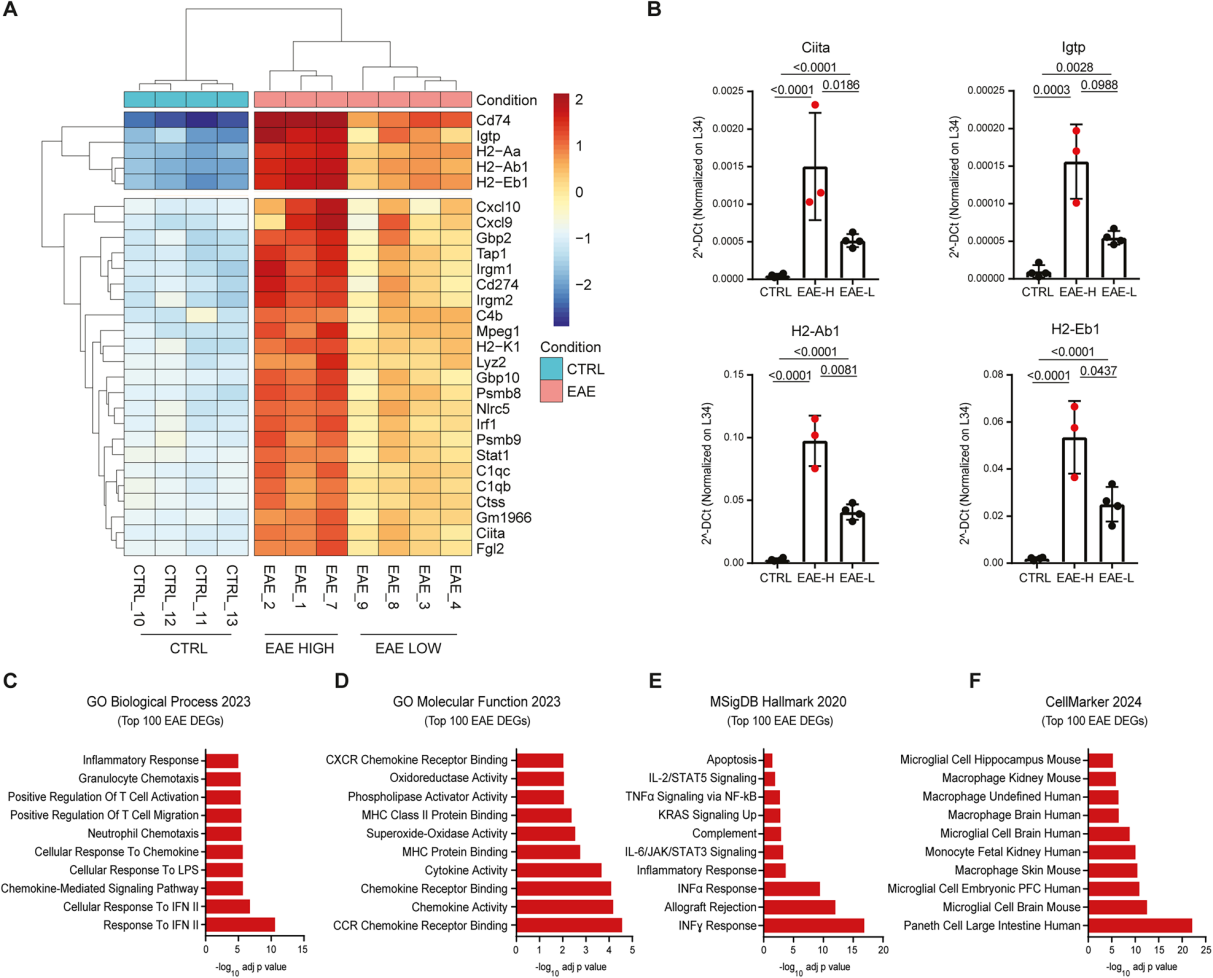
Transcriptome analysis identifies EAE subgroups characterized by high and low inflammation of the prefrontal cortex. **A** Heat map representation of the top 100 differently expressed genes (DEGs) genes in the PFC of the control (CTRL) (blue) and EAE-mice (orange). The colour legend indicates the scale of gene expression changes detected (red=upregulated; blue=downregulated). The clustering dendrogram shows the presence of two EAE subgroups, one showing higher differences compared with the CTRL samples (EAE 1, 2 and 7: EAE-H) than the other (EAE 3, 4, 8, 9: EAE-L). **B** Bar graphs showing significantly higher changes of expression levels of representative inflammatory genes (*Ciita, Igtp, H2-Ab1, H2-Eb1*) in the EAE-H subgroup compared to the EAE-L and/or CTRL groups (One-way ANOVA, *p* < 0.05; Tukey post hoc comparisons *p* < 0.05). All values are given as means ± SD. **C** Biological process, (**D**) Molecular function, (**E**) MSig DB Hallmark 2020 and (**F**) Cell marker 2024 annotations of the top 100 differently regulated genes in the PFC of EAE mice compared with CTRLs.

Unbiased clusterization of the most significant DEGs (*n* = 100; Additional Table 3) indicated the presence of two EAE transcriptional states, one showing higher differences with the CTRL samples (EAE 1, 2 and 7) and the other displaying lower or intermediate changes (EAE 3, 4, 8 and 9) (Fig. 2A; Additional file 3). Analysis by qPCR confirmed that the genes mostly contributing to the subclustering of EAE samples (i.e. *H2Ab1, H2Eb1, Ciita, Igtp*) were expressed at significantly higher levels in the PFC of the former EAE group compared with both controls and the latter EAE subgroup (Fig. 2B). These top-ranking DEGs are strongly related to inflammatory pathways and responses to cytokine signaling (Fig. 2C–E). Significant enrichment was also observed for genes of the Major Histocompatibility Complex type II (MHC-II), like *Cd74*, *H2Eb1* and *H2Ab1* (Fig. 2A, D), which are involved in the antigen presentation response pathway [36]. Genes involved in the interferon response (*i.e. Cxcl10*, *Gbp2*) or encoding for proteins of the complement system (i.e*. C1qb*, *C1qc, C4b*) were also particularly enriched (Fig. 2A, E). Furthermore, the query of the Cell Marker database, which provides a manually curated collection of cell type markers [37], indicated an enrichment of genes that characterize myeloid cells, such as macrophages and microglia (Fig. 2F). Thus, based on the transcriptional levels of these inflammatory genes, we subdivided the EAE mice into high (H) and low (L) inflammation subgroups (Fig. 2A).

### Changes in expression levels of MS-related genes in the PFC is associated with increased inflammation

**Fig. 3:**
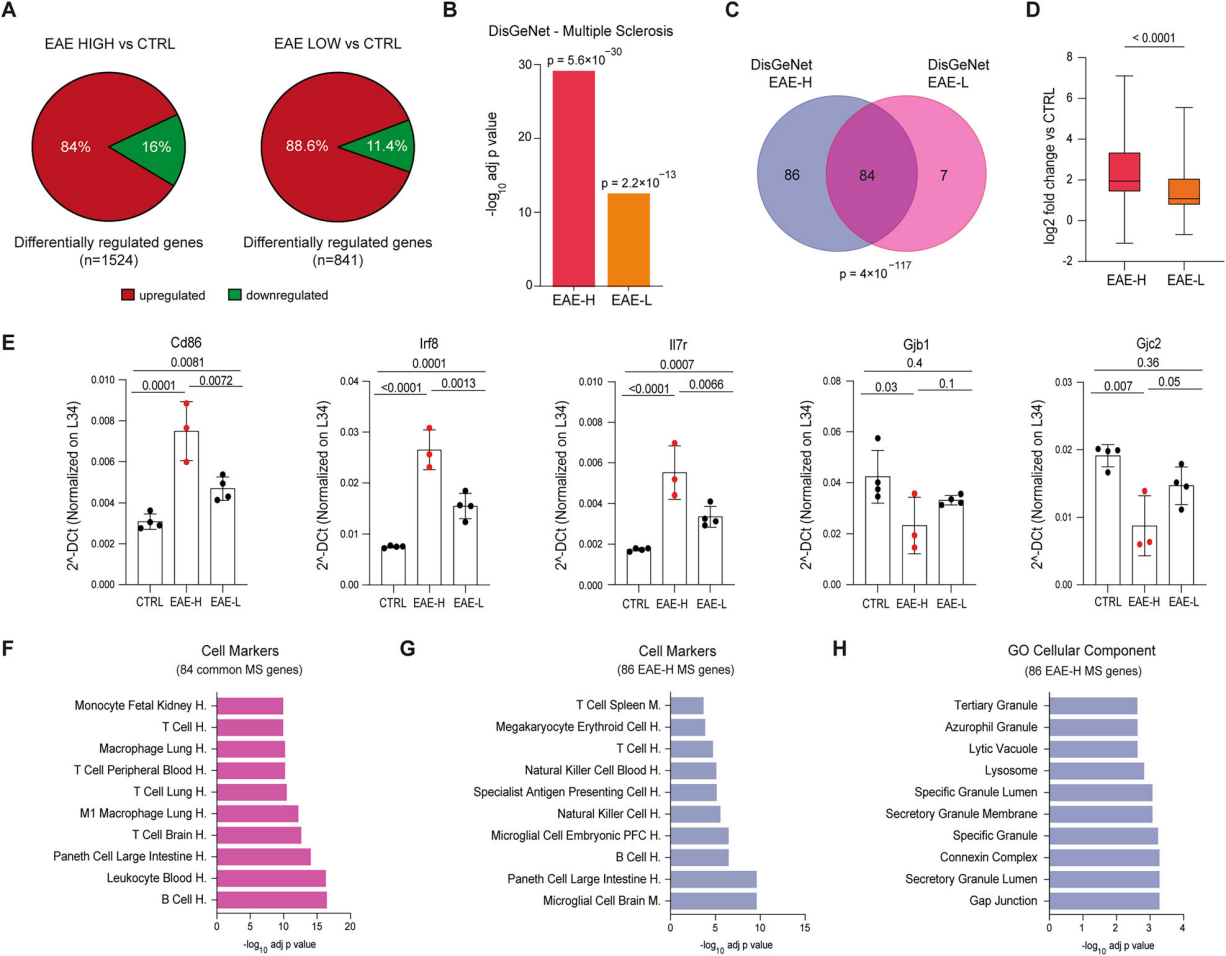
High inflammation leads to widespread alteration of MS-associated genes in the prefrontal cortex (PFC). **A** Pie charts showing upregulated (red) and downregulated (green) genes in the PFC of EAE-H and EAE-L mice. **B** Annotation of differently expressed genes (DEGs) in the DisGeNet catalogue database performed using the ENRICHR tool (https://maayanlab.cloud/Enrichr/enrich) in both EAE subgroups. **C** Venn diagrams of MS-associated lists of genes from the DisGeNet database showing overlap between the EAE-H and EAEL subgroups. **D** Boxplots showing distribution and statistical significance of fold change variations of reads related to DEGs in EAE-H and EAE-L subgroups vs controls (CTRL). **E** Bar graphs showing qPCR analyses of the indicated representative MS-associated genes that are common to both subgroups of EAE samples (One-way ANOVA, Tukey post hoc test, *p* < 0.05). All values are given as means ± SD. **F**, **H** Cell Markers annotations for MS-associated DEGs common to both subgroups (**F**) and genes exclusively modulated in the EAE-H subgroup (**G**). **H** Cellular component annotations of MS-associated genes showing changes exclusively in the EAE-H subgroup.

To further elucidate the disease-associated transcriptional changes occurring in the PFC of EAE mice, we separately analyzed the EAE-H and EAE-L groups. The CSS score at sacrifice, which indicates the disease severity at the time of the transcriptomic analysis, was not significantly different between EAE-H and EAE-L mice (Additional file 4A). However, EAE-H mice tended to reach the peak of disease earlier than the EAE-L mice (16 d.p.i. vs 19.5 d.p.i.), albeit this difference was not statistically significant (Additional file 4B, C). When compared to control mice (Additional file 4D, E), the EAE-H PFC showed a higher number of DEGs than that of EAE-L mice (Fig. 3A, Additional Table 4). To evaluate whether the transcriptional signatures of the EAE-H and EAE-L PFC are representative of human disease states, we queried the DisGeNet database [31]. Genes associated with MS and other autoimmune diseases were enriched among the DEGs of both EAE subgroups (Additional file 4F, G, Additional Table 5). However, the EAE-H PFC displayed a higher statistical significance and a higher number of MS-related DEGs compared to the EAE-L PFC (Fig. 3B, C). Furthermore, genes common to the two subgroups were significantly more deregulated in the EAE-H PFC (Fig. 3D, E, Additional Table 5). These observations support the reliability of this model for the study of cortical damage in MS and suggest that the level of inflammation in the PFC can differentially affect the regulation of MS-related genes. In support of this notion, we noted that, while MS-related genes whose expression was altered in the two subgroups (*n* = 84) are mainly enriched in markers of B and T cells (Fig. 3F), MS-related DEGs that are specific of the EAE-H PFC (*n* = 86) were enriched in markers of mouse brain microglia and of human fetal PFC (Fig. 3G), indicating the involvement of additional cell types and/or functional activities. Moreover, gap junction proteins associated with both peripheral and central demyelinating diseases, such as *Gjb1* and *Gjc2* [38, 39], were specifically downregulated in the EAE-H PFC (Fig. 3E, H; Additional Table 5). Thus, our data suggest that mice with similar CSS scores may display variable involvement of the PFC and that increased inflammation in this area significantly impacts on the expression of MS-associated markers of both resident (microglia) and nonresident (B and T lymphocytes) immune cells.

### EAE-H mice feature an increased impact of local inflammation on cells of the PFC

**Fig. 4:**
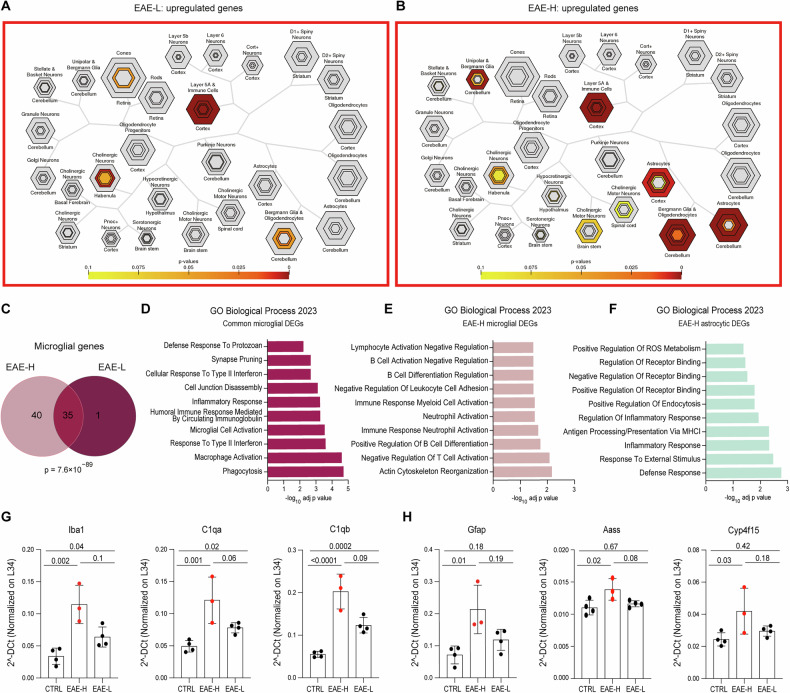
Cell-specific expression analysis (CSEA) of upregulated genes in both EAE subgroups. CSEA (http://doughertytools.wustl.edu/CSEAtool.html) of upregulated genes in the EAE-L (**A**) and EAE-H (**B**) subgroups. Each cell type is represented by different concentric hexagons. Varying stringencies for enrichment (pSI) are represented by the size of the hexagons, with outer hexagons representing the least significant lists and central ones the most significant (Xu et al., 2014). The overlap of candidate gene lists for each cell type is calculated with Fisher’s Exact Test: *p* > 0.05 and is represented in light orange/yellow, while *p* < 0.05 is represented in dark red. **C** Venn diagram of microglia-associated lists of genes in the EAE-H and EAE-L subgroups obtained with the CSEA tool. Annotations of Biological Process of microglial upregulated genes common to both subgroups (**D**), microglial genes exclusively upregulated in the EAE-H subgroup (**E**), and astrocytic genes (**F**), which are differently regulated only in the EAE-H subgroup. Bar graphs showing qPCR analyses performed for validation of representative genes specific for microglia (**G**) or astroglia (**H**), showing higher expression levels in the EAE-H group (One-way ANOVA, Tukey post hoc test, *p* < 0.05). All values are given as means ± SD.

To investigate which of the two EAE phenotypes affected CNS cells in the PFC, DEGs were queried by the Cell Type-Specific Expression Analysis (CSEA) tool (http://doughertytools.wustl.edu/CSEAtool.html). The genes upregulated in the EAE-L PFC mainly overlapped with the immune cell category (Fig. 4A), potentially represented by microglia [40]. However, the EAE-H PFC also showed significant upregulation of genes overlapping with astroglial categories (Fig. 4B), which contribute to the severity of the EAE phenotype [41]. Moreover, a higher number of microglia-related genes was altered in the EAE-H PFC compared with the EAE-L PFC (Fig. 4C). GO analysis of the common microglia genes (*n* = 35) highlighted biological processes related to microglia activation and acquisition of an activated phenotype, such as phagocytosis, inflammatory response and synapse pruning (Fig. 4D). On the other hand, microglia genes uniquely affected in the EAE-H PFC were associated with actin cytoskeleton reorganization and with nonresident immune cell activation (i.e. neutrophils, B and T lymphocytes) (Fig. 4E), suggesting recruitment of infiltrating peripheral cells and their participation to the inflammatory process in this brain area of EAE-H mice. Astrocytic genes, whose expression is specifically changed in the EAE-H PFC, were associated with the response to external cues and with the inflammatory response (Fig. 4F). This observation suggests the acquisition of a reactive phenotype by astrocytes and their involvement in local neuroinflammation [42]. Validation by qPCR analysis confirmed the increased or specific regulation in the EAE-H PFC of, respectively, microglia (*Iba1*, *C1qa*, *C1qb*) and astroglia (*Gfap*, *Aass*, *Cyp4f15*) genes (Fig. 4G, H). Since microglia activation anticipates that of astroglia during CNS inflammation [43], these observations confirm the more advanced stage of PFC neuroinflammation in EAE-H mice.

Next, we evaluated differences among the genes that are downregulated in the PFC of the two EAE subgroups. The CSEA tool indicated that neuronal genes were either more significantly (layer 5b, layer 6 neurons) or exclusively (cortical neurons) enriched among the EAE-H downregulated genes (Additional file 5A–C). These EAE-H specific neuronal genes (*n* = 26) are related to synaptic functions, such as chemical transmission, neuron projection and dendrites (Additional file 5D, E), and they encode for proteins of potential relevance for CI in neurodegenerative diseases, such as BDNF, the interneuron markers pronociceptin (PNOC) [44] and somatostatin (SST) [45], the neuronal differentiation factor NEUROD6 [46] and the synaptic proteins NPTX2 [47] and HOMER1 [48]. Moreover, genes associated with oligodendrocytes, which are typically damaged in MS and EAE [29], were enriched only among the EAE-H downregulated genes (Additional file 5B). Interestingly, these oligodendrocyte genes are significantly associated with CNS demyelinating diseases in the DisGeNet database (Additional file 5F). Analysis by qPCR confirmed the augmented (*NeuroD6*, *Pnoc*) or selective (*Gjb1*, *Gjc2, Sst*, *Slc45a3*) regulation of these genes in the EAE-H PFC (Fig. 3E, Additional file 5G). Thus, increased inflammation in the EAE-H PFC also leads to changes in the transcriptome of neurons and oligodendrocytes, with possible consequences on the functions of these cell types.

### Markers of activated microglia and astroglia are differentially expressed in the PFC of EAE-H and EAE-L mice

**Fig. 5:**
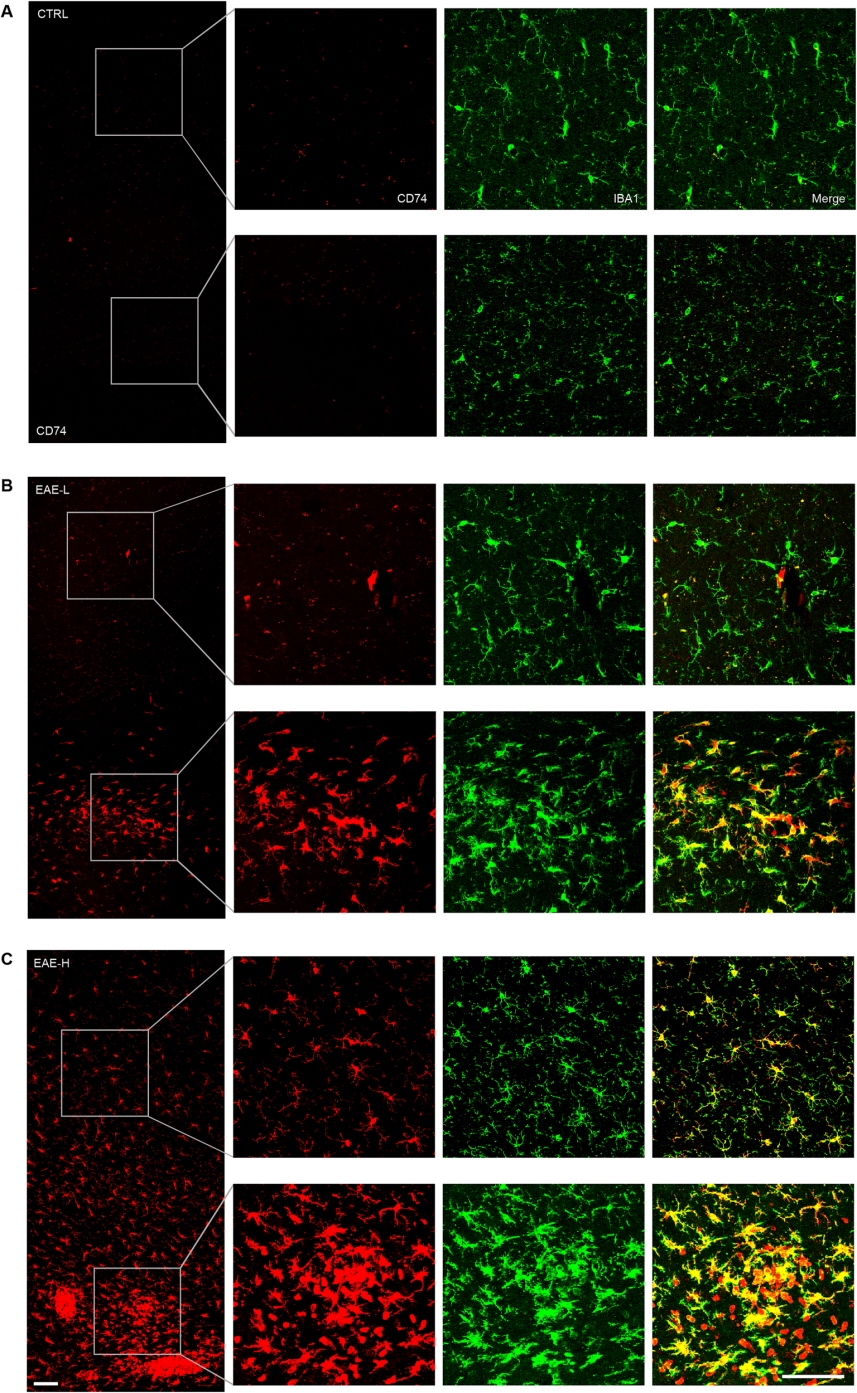
EAE-H mice exhibit a wider expression of microglial CD74 compared to the EAE-L subgroup. Representative confocal microscope immunofluorescence images of CD74 (red)/Iba1 (green) double-stained PFC coronal sections from controls (CTRL (**A**), EAE-L (**B**) and EAE-H mice (**C**). EAE-H mice exhibit a strong CD74 immunostaining in both inflammatory lesions located in the deep PFC white matter and parenchymal microglia, which extends to regions distant from the infiltrating area, reaching the subpial surface. However, in EAE-L animals, CD74 staining is limited to the lesion site. Scale bar 100 µm.

To identify which neural cell type primarily contributed to the production of molecules whose expression is responsible for the subdivision into EAE-H and EAE-L subgroups, we investigated the co-expression of DEGs genes with markers of microglia (Iba1) or astrocytes (GFAP), glial cells known to respond to inflammatory cues in the CNS. To this end, we selected the top-scoring gene *Cd74* and the complement factor *C1q*, which are known to be upregulated in monocytes/microglia and astrocytes during MS and EAE [33, 49–51]. Confocal analysis of Iba1^+^ cells indicated the increased presence of microglia in the PFC of both EAE subgroups with respect to control samples, with significantly higher abundance of these resident immune cells in the EAE-H PFC (Additional file 6A–D). In addition, CD74 staining appeared mainly co-localized with Iba1^+^ microglia/macrophages (Fig. 5A–C), whereas limited staining was observed in GFAP^+^ astrocytes (Additional file 6E–G). Importantly, while CD74 immunoreactivity was widely distributed in the EAE-H PFC, where it is detectable also in cells distant from the inflammatory lesions (Fig. 5C), its expression was restricted to cells clustered close to the lesions in the EAE-L PFC (Fig. 5B). Likewise, we observed that C1q protein was selectively expressed by amoeboid Iba1^+^ microglia/macrophages that are localized near the inflammatory infiltrates in the PFC of EAE-H mice, whereas it was barely detectable or absent in the EAE-L and control PFC (Additional file 7A, B). Our in-silico analyses also predicted the increased involvement of astrocytes in the EAE-H PFC (Fig. 4A, B). In line with this notion, we observed extensive staining for the astrocyte marker GFAP in these mice (Additional file Fig. 7C–E). Together, these results suggest that the presence of a microglia subset characterized by expression of MHC-II and complement proteins in the PFC is associated with increased inflammation during the acute phase of EAE.

### High expression of markers of microglia activation in the PFC correlates with impaired cognitive functions in EAE mice

**Fig. 6:**
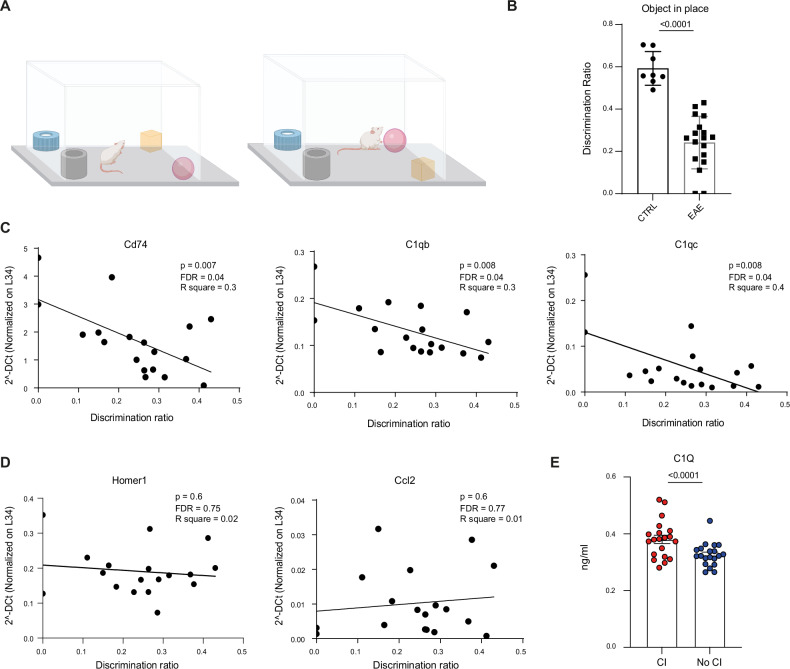
Expression of specific inflammatory markers correlates with cognitive performances in EAE mice. **A** Schematic representation of the Object in Place (OIP) test. **B** Bar graphs show the discrimination ratio scored by control (CTRL; *n* = 8) and EAE (*n* = 18) mice in the OIP test. A highly significant reduction is evident in EAE mice compared to CTRLs (Student’ s *t* test, *p* < 0.0001). **C**, **D** Pearson’s correlation analyses of the expression levels of selected genes in the PFC of EAE mice and the discrimination ratio scored by the same mice in the OIP test. While a significant inverse correlation is evident for the inflammatory genes *Cd74*, *C1qb* and *C1qc* (C), no significant correlation is detectable between other inflammatory markers, such as *Ccl2*, or the neuronal gene *Homer1* (**D**). **E** Bar graphs point out the significantly increased expression of C1q protein level in the CSF of MS patients showing Cognitive impairment (CI) compared with MS patients not showing CI (Student’s *t* test, *p* < 0.0001). All values are given as means ± SD.

Electrophysiological and behavioural evidence suggests a functional contribution of PFC to recognition memory [52]. Coherently, damage in this region was shown to impair memory tasks for object location [53]. Therefore, to explore whether EAE-H PFC markers were associated with CI, we employed a separate cohort of control (*n* = 8) and EAE (*n* = 18) mice. Cognitive evaluation was performed by the OIP test at the presymptomatic phase of the disease (10–14 d.p.i.), whereas inflammatory markers were evaluated by qPCR at the peak of the acute phase (CSS = 1.75–2.5), when mice were sacrificed (Fig. 1A). EAE mice scored significantly lower in the OIP test, albeit with considerable variation in the extent of their functional impairment (Fig. 6A, B). Notably, there was no correlation between CI at this presymptomatic stage and the severity of EAE, as determined by the CSS value at the peak of the acute phase (Additional file 8A). Furthermore, while all the inflammatory genes tested were significantly upregulated in the PFC of EAE mice (Additional file 8B, C), they displayed variable correlation with CI in the OIP test. For instance, expression levels of *C1qb*, *C1qc*, *Cd74* and other MHC-II (*H2Ab1*, *H2Eb1*) or chemokine (*Ccl5*) genes were negatively correlated with cognitive performance, whereas others (i.e., *H2Eb1*, *Ccl2*, *Ccr2*, *Saa3*, *Tnf*), were not (Fig. 6C, D, Additional file 8B, C). Moreover, no significant correlations between the performances of EAE mice in the OIP test and expression levels of the neuronal genes *Homer1*, *Nptx2*, *Pnoc* and *Bdnf* were evident (Fig. 6D, Additional file 8C).

Risk allele variants of genes encoding for C1q proteins were associated with impaired resolution of inflammation in MS patients [50]. Moreover, the complement system was shown to be involved in EAE pathology in mice [54], whereas conditional knockout or pharmacologic inhibition of C1q function reduced EAE-associated gliosis [50]. Thus, since C1q proteins are secreted in body fluids, we further focused on their possible role as biomarkers of CI. To this end, we selected a cohort of MS people with (CI^+^, *n* = 20*) or w*ithout (CI^-^, *n* = 20) overall CI (see Methods), as assessed by formal neuropsychological evaluation performed at the time of lumbar puncture and disease diagnosis. Despite the two cohorts did not differ in terms of demographic and clinical characteristics (Additional Table 6), analysis by ELISA of cerebrospinal fluid (CSF) samples demonstrated a significantly higher level of C1q in the CI^+^ group compared to the CI^-^ group (Fig. 6E). These results indicate that activation of a specific inflammatory signature in the PFC microglia correlates with CI in EAE mice and suggest that C1q levels in CSF may represent a suitable pathophysiological marker associated with CI in MS patients.

## Discussion

MS is a complex disease characterized by deficits in multiple functional systems and neurodegenerative changes in grey matter structures, which are associated with both psychiatric disturbances and impairment of cognitive functions [7, 19]. The PFC is deeply involved in cognition [55] and mounting evidence points to an important role of PFC dysfunction in MS-related CI [12, 15–17, 56]. Nevertheless, a comprehensive analysis of gene expression changes induced by neuroinflammation in this cortical area of MS patients or animal models is still lacking. To fill this gap, we have performed genome-wide transcriptomic analysis of the PFC of SJL/J EAE mice, a model of MS characterized by extensive cortical neuroinflammation and signs of CI that are already manifested at the presymptomatic phase of the disease [9, 21, 22]. Our analysis uncovered a widespread transcriptome reprogramming triggered by neuroinflammation in the PFC. Genes induced by EAE were associated with inflammation, immune cell responses and autoimmune diseases, including MS. On the other hand, repressed genes were associated with neuronal functions and psychiatric or neurological syndromes. These findings point to an early involvement of the PFC in EAE and support the suitability of the EAE SJL model for the study of cortical lesions in MS.

Our study revealed that EAE mice displaying similar CSS can differ in the inflammatory status of the PFC and that this latter parameter, but not the CSS, correlates with the occurrence of CI. This observation may reflect the different involvement of brain regions engaged in motor and cognitive functions. Indeed, CSS is mainly assessed by monitoring motor functions and is likely influenced by EAE-induced inflammatory processes occurring in the spinal cord. Thus, our findings indicate that inflammation in the PFC represents an early sign and a possible marker of CI risk. The variable involvement of the PFC in individual EAE mice is consistent with the heterogeneity of inflammatory patterns, pathological features, and clinical course described in both MS patients [57, 58] and EAE mice [9, 59]. Interestingly, among the genes that mostly distinguished the EAE-H and EAE-L subgroups, we found several associated with the microglia. Since microglia participate in the extensive remodelling of neuronal contacts that occur during development by promoting synaptic pruning [60], it is possible that increased expression of these genes in the EAE-H PFC may reflect the occurrence of aberrant synaptic pruning that disrupts the established neuronal circuitry, leading to reduced cognitive performance.

Higher levels of inflammation in the EAE-H PFC were associated with wider changes in the expression of MS-associated genes. Besides inflammatory genes, we noticed the modulation of genes involved in autoimmunity and genes related to disease susceptibility, like *Il7r*. Moreover, expression of the connexins CX32 and CX47 was downregulated in the EAE-H PFC. These proteins are involved in gap junction communications between oligodendrocytes and astrocytes [61], and mutations in their genes cause demyelinating pathologies, like Charcot–Marie–Tooth disease [39] and Pelizaeus– Merzbacher-like disease [38]. Taken together, these results suggest that the progressive worsening of inflammation causes the parallel engagement of molecular pathways relevant to different pathogenic aspects of MS. Moreover, high levels of PFC inflammation are associated with a more advanced tissue response to damage. In fact, modulation of both oligodendroglial and neuronal genes was detectable only in the EAE-H PFC. This finding suggests that increased inflammation is paralleled by more severely damaged tissue. Several downregulated neuronal genes are of direct relevance for CI. For instance, *Pnoc* encodes nociceptin, a peptide produced by a subset of interneurons involved in stress responses, pain transmission and reward [62]. Likewise, SST is known to play a crucial role in memory and cognition [63]. Altered expression of interneuron-specific genes is in line with our previous observation that EAE induces changes in GABAergic circuitry in the PFC [9]. Notably, the *Pnoc* and *Sst* transcripts were previously shown to be downregulated in the PFC, also in response to LPS-induced neuroinflammation [26] or to physiological ageing [64]. In addition, reduced levels of SST protein were found in the CSF of MS patients [65]. We also observed downregulation of genes encoding proteins of excitatory synapses, such as HOMER1 [48] and NPTX2 [47], which may also be relevant for CI. Indeed, NPTX2 was proposed as a CSF biomarker of disease progression in frontotemporal dementia [66]. Together, these observations indicate that increasing levels of inflammation gradually affect multiple neuronal phenotypes in the PFC and negatively impact the expression of neuronal genes that are associated with CI in other pathologies.

Microglia represent the first line of defence in the CNS [67]. While upregulation of microglial genes occurred in both subgroups, the EAE-H PFC featured induction of a higher number of genes, including markers of activation, such as *Cd68*. Moreover, virtually all Iba1^+^ cells in the EAE-H PFC expressed the CD74 protein, further indicating the acquisition of an activated phenotype [49]. Recent findings demonstrated that active lesions in human MS brain are characterized by a specific subset of microglia expressing high levels of MHC-II related genes, including CD74 [59]. This microglia subset featured higher expression of several inflammatory markers, indicating its contribution to the propagation of damage in these brain lesions [59]. In addition, expression of *Cd74* and other MHCII genes was shown to label a subset of microglia involved in antigen presentation, exhibiting marked interactions with encephalitogenic T cells [68]. We found that CD74^+^ microglia were also diffused in regions that are distant from the lesion sites in the EAE-H PFC, which is reminiscent of the signs of inflammatory reaction in the absence of plaques observed in normal-appearing white matter of MS people [69]. The diffuse inflammatory process throughout the PFC of these mice may also be analogous to the smoldering inflammation that causes widespread tissue damage and promotes progressive physical disability and CI in MS patients [70–72].

Our data indicate that PFC inflammation, but not CSS values, is correlated with early occurring CI in EAE mice. This finding is in line with the absence of clearcut relations between physical disability and CI described in people with MS [73]. Thus, we speculate that PFC inflammation may represent an early pathophysiological event associated with CI. This hypothesis is corroborated by the significant inverse correlation shown between the expression levels of specific inflammatory markers, such as MHC-II and complement factors, and performance in the cognitive test. MHC-II proteins are upregulated in resident and infiltrating myeloid cells in both MS [74, 75] and other neurodegenerative diseases characterized by CI, such as AD [76]. There is also evidence supporting the association between upregulation of complement system molecules and CI. Complement activation is associated with glial activation, aberrant synaptic pruning and neuron loss in neurodegenerative diseases associated with neuroinflammation and progressive CI, such as AD, as well as in physiological ageing [77]. In this regard, a recent study showed that C1q produced by microglia during ageing is internalized by neurons and integrated into ribonucleoprotein complexes, which affect protein synthesis, neuronal plasticity and, possibly, CI [78]. We found that C1q proteins are expressed by activated hypertrophic microglia/macrophages surrounding inflammatory infiltrates in the EAE-H PFC. Complement over-activation during MS course has been described in both white matter and grey matter lesions [79–81]. At the cortical level, C1q expression increases in regions exhibiting high numbers of complement receptor-positive microglia [80], while it was found close to lesions showing active inflammation in the thalamus [81], thus reinforcing the hypothesis of its contribution to the worsening of the inflammatory damage during MS progression [79, 80]. Indeed, high expression of C1q-encoding genes characterizes a subpopulation of “inflamed microglia” associated with active lesions in MS brain [50]. Moreover, activation of the complement system was proposed to contribute to the propagation of inflammatory damage at the lesion edge [50]. In our setting, expression of the *C1qb* and *C1qc* genes in the EAE PFC was significantly correlated with CI. Even more importantly, we found a significant increase in C1q proteins in the CSF of MS patients exhibiting CI. This novel result critically reinforces the hypothesis that microglia and the complement system might be involved in the pathogenesis of MS-related CI, potentially through an effect on surrounding neurons and synapses. To date, studies on CSF markers that correlate with CI in MS are sparse and not univocal. Data exist on the association between markers of axonal damage and deficits of information processing speed [82], and between markers of B cells activation and cortical functions, particularly verbal memory deficits in MS [83]. Our results indicate, for the first time, that the complement system might also be a valuable biofluid marker reflecting the presence of global CI. They also highlight recent evidence that intrathecal complement activation is associated with radiological and molecular biomarkers reflecting MS pathophysiology [84]. Further work will be needed to explore the possible correlation between CI, the expression of C1q proteins and that of established markers of neuronal injury, synaptic dysfunction or microglial/astrocytic activation. These studies will help to better define the contribution and relative weight of the complement system in the pathogenesis of this disabling manifestation of the disease.

In conclusion, our study uncovers the link between increased PFC inflammation and CI in the EAE model and identifies inflammatory genes, such as C1q, as early markers or risk factors for functional impairment of the PFC. These findings could pave the way for the development of personalized approaches to the diagnosis and management of CI in MS patients.

## Materials and methods

### Immunization and EAE induction in SJL/J mice

Mice coming from different litters and cages were randomly assigned to the different experimental groups. EAE was induced in female SJL/J mice (8–10 weeks old; The Jackson Laboratory, Bar Harbor, ME; *n* = 28) as previously described [9, 22, 85]. Briefly, mice were subcutaneously injected on day postimmunization (d.p.i.) 0 and 7 with 100 μl of an emulsion containing 75 μg of PLP_139-151_ (PRIMM, Milan, Italy) and enriched complete Freund’s adjuvant (CFA) with 8 mg/ml of killed and heat-dried *Mycobacterium Tuberculosis* H37RA (Sigma-Aldrich S.r.l., Milan). Intraperitoneal administration of 300 ng *Bordetella Pertussis* toxin (Merck, Milan, Italy) was performed on d.p.i. 0, 1, 7 and 8 [9]. Clinical Score and Signs (CSS) of EAE were daily evaluated as previously described [9, 86].

CSS was evaluated blindly by two different experimenters and the mean value of their scores was used by the data collector.

Control mice (*n* = 12) were injected only with CFA. EAE mice were euthanized at the peak of the acute phase of the disease (CSS = 1,75–2,5; d.p.i 17 ± 2), and control mice were euthanized on the same days for comparison. We excluded animals that failed to develop proper symptoms (*n* = 1) or underwent remission in the 48 h preceding the sacrifice (*n* = 2).

Mice were perfused with sterile saline solution under deep anesthesia (87.5 mg/Kg Ketamine + 12.5 mg/Kg xylazine; 0.1 ml/20 g mouse weight), the brain was removed and one hemisphere was fixed in 4% paraformaldehyde for 48 h and employed for morphological analysis, while the PFC extracted from the other hemisphere was used for molecular assays and processed accordingly.

### Object in place (OIP) task

8–10-week-old SJL/J female control (*n* = 8) and EAE (*n* = 18) mice were analyzed by the OIP assay. Mice were recorded by a camera located directly above the apparatus and scored blindly. The test was carried out at the presymptomatic phase of EAE (10 ± 2 d.p.i.), when the EAE mice did not show motor deficits yet [9], with a CSS = 0,5 ± 0,25 essentially related to mild loss of tail tone (Additional file1A). The test was performed as described [52]. 24 h before the test, we assessed a pre-training phase, in which all mice were habituated to the empty arena for 15 min.

First, in the acquisition phase, each animal was located in the centre of a rectangular arena, where four different objects had been placed at the corners, at a distance of 15 cm from the walls. Each mouse was allowed to investigate the objects for 5 min before being removed and placed in the home cage for 5 min (delay phase). During this interval, after cleaning with alcohol, two of the objects were exchanged in position. Then, in the test phase, each animal was placed again in the arena and was allowed to explore the objects for 5 min [52]. Discrimination ratio (the ratio between time spent with the novel object and the total time spent exploring the two objects) and total exploration time (total time spent exploring the two objects) were calculated. The experiment was replicated in two separate cohorts of mice.

### RNA isolation and quantitative real-time PCR analyses

The PFC of control and EAE mice were isolated and maintained in RNA-later stabilization reagent (QIAGEN). Total RNA was extracted, and DNase treated using the RNAeasy Mini Kit (QIAGEN), according to the manufacturer’s instructions. RNA purity and concentration were quantified using the NanoDrop 2000 UV spectrophotometer (Thermo Scientific). Total RNA (1 μg) was reverse-transcribed with random primers (Sigma-Aldrich) using M-MLV reverse transcriptase (Promega), according to manufacturers’ instructions. Quantitative real-time PCR (qPCR) analyses were performed to validate RNA-seq results in 3 separate cohorts of mice. Briefly, 15 ng of cDNA were used as template. The qPCR reactions were performed using the LightCycler 480 System (Roche) with SYBR Green I Master Mix (Roche) as previously reported [9, 85]. The 2 − ΔCt method was applied to calculate differences in the gene expression using the L34 gene for data normalization and quantified as previously described [9, 85]. Primers used for qPCR analyses are listed in Additional Table 1.

### RNA-sequencing and bioinformatics analyses

RNA-sequencing (RNA-seq) was performed on control (*n* = 4) and EAE (*n* = 7) mice, using a 150 bp paired-end format on an Illumina NovaSeq6000 as previously described [87, 88]. We used Sequencing, data quality, reads repartition, and insert size estimation were performed using FastQC v0.11.9, Samtools v1.9 (http://www.htslib.org/), Picard v2.26.6 (Picard Toolkit, Broad Institute) and RSeQC v.4.0.0. The raw pair-end reads were processed to remove the adapters with cutadapt v.3.1 and corrected for reads quality and length through fastp v.0.21.0. The reads were then mapped to the mouse genome (mm10/GCRm38) with Gencode (vM25) gene annotation using STAR aligner v.2.7.9a. The uniquely mapped reads were kept and counted using feature Counts v.1.6.0 (http://subread.sourceforge.net/). Based on these read counts, normalization and differential gene expression were performed using DESeq2 (v.1.16.0 on R v.3.1.3).

Results were considered statistically significant for *p* values ≤ 0.05 and fold-changes ≥ 1.5.

### Functional enrichment and computational analyses

Gene Ontology (GO) analyses were performed using the TopGo package in R Studio Software. All analyses were performed using the total number of genes expressed in both experimental groups as background. Overlap analyses were performed using Venny 2.1.0 software and the significance was assessed by hypergeometric test using the phyper function of R StatsPackage in R Studio Software. DisGeNet analyses were carried out using the Enrichr tool (https://maayanlab.cloud/Enrichr/). Gene Set Enrichment Analysis (GSEA) was performed using the GSEA software version 4.3.2 (https://www.gsea-msigdb.org/gsea/index.jsp) [89]. A comparison of the gene expression pattern between EAE and control mice was conducted using DEGs derived from the DESeq2 analysis of the RNA-seq dataset. Pre-ranked GSEA was conducted using gene lists whose ranking was based on the log_2_(fold change) value. Parameters for accepted gene sets were *p* < 0.05 and false discovery rate (FDR) < 0.05.

### Immunofluorescence

After fixation and cryoprotection in sucrose 30%, 30 µm thick serial coronal PFC sections were cut with a microtome, as previously described [9, 85]. Every sixth section was stained with Nissl or Neuro-Trace (Thermo Fisher Scientific, Waltham, MA USA) staining for histologic analysis or processed by immunocytochemistry for the following antigens: rabbit anti-Iba1 (Wako, Richmond, VA, USA; 1:1000), goat anti-Iba1 (Novus Biological, Centennial, CO, USA; 1:500); rat anti-mouse CD74 (BD Phamingen^TM^, New Zeland, 1:1000), rabbit anti-C1q (Abcam, USA, 1:100), rabbit antiGFAP (Agilent, USA; 1:1000), mouse anti-GFAP (Santa Cruz Biotechnology, Inc. Texas, USA; 1:500). Reactions were revealed using the following secondary antibodies: FITC-anti-rabbit IgGs (1:200; Vector, Burlingame, CA, USA), cyanine-3-(Cy3)-conjugated IgGs (donkey anti-rat, anti-goat or anti-mouse Cy3-IgGs) (Jackson Immunoresearch Laboratories, West Grove, PA, United States; 1:200) and counterstained with DAPI (Thermo Fisher Scientific, Waltham, MA USA) [9]. Confocal laser scanning microscope (Nikon Ti2) was employed to analyze the localization of the markers investigated. Immunofluorescence experiments were performed in samples from 3 different cohorts of mice.

### Quantitative evaluation of Iba1 immunoreactivity

Semiquantitative evaluation of Iba1 immunoreactivity in the PFC of control, EAE-H and EAE-L mice (*n* = 3/each group) was performed by densitometric analysis, as previously described [25]. Z-stack confocal microglia images were acquired at 20X magnification in 1-in-12 series of PFC sections (34 section/animal), then exported in TIFF and analyzed with the ImageJ software (https://rsb.info.nih.gov/ij/; National Institutes of Health, Bethesda, MD, USA). For each image, a background threshold was determined by measuring the mean grey value in a non-stained area and applied uniformly across all sections. The threshold was manually set and kept constant between groups. Regions of interest (ROIs) were drawn over defined tissue areas and mean grey value (F) and area (A) were measured. Fluorescence was expressed as F/A, representing the mean signal intensity normalized to tissue area.

### Human samples and cognitive impairment assessment

We retrospectively selected 40 people with MS who underwent CSF analysis as part of their usual MS diagnostic work-up from January 2018 to January 2020 at the University Hospital of Perugia (Italy). All patients signed an informed consent and were assessed by neurologists with experience in the management of MS. Demographical and clinical data were anonymously collected in an electronic database. CSF samples were collected via lumbar puncture, using the same standard operating procedures throughout the study [90]. CSF was collected as part of the usual MS diagnostic work-up, aliquoted, and immediately frozen at −80 °C, together with serum aliquots. MS patients underwent a neuropsychological evaluation within 30 days by CSF collection.

For patients’ selection, we applied the following inclusion criteria: (i) a diagnosis of RRMS according to the 2017 revision of the McDonald criteria [91]; (ii) age > 18 years; (iii) no history of learning disability or drug or alcohol abuse. Patients were selected to achieve a 1:1 representation of individuals with and without CI, as determined by a standardized neuropsychological evaluation performed by a trained neuropsychologist by using Rao’s Brief Repeatable Battery of Neuropsychological Tests (BRBN) [92]. The BRBN explores: (i) verbal learning and delayed recall [Selective Reminding Test (SRT) Long-Term Storage, SRT Consistent Long-Term Retrieval and SRT Delayed Recall]; (ii) visuospatial learning and delayed recall [10/36 Spatial Recall Test (SPART) and SPART Delayed Recall]; (iii) IPS [Paced Auditory Serial Addition Test (PASAT) 2 and PASAT-3, and Symbol Digit Modalities Test]; and (iv) verbal fluency on semantic input (Word List Generation). A test score was considered altered when lower than the 5th percentile [93]. The presence of overall CI was defined by the impairment of at least two cognitive domains [94]. Subjects without CI were selected for the absence of impairment in any of the tested domains.

### ELISA assays

CSF from MS patients was thawed, centrifuged to remove any debris and immediately processed to measure C1qB content through the Double antibody-Sandwich ELISA (FineTest, Wuhan, 430074, Hubei, China), according to the manufacturer’s instructions (sensitivity 0.094 ng/ml). The absorbance values at 450 nm (A450 nm) of unknown samples were always within the linearity range of calibration curves, drawn with increasing concentrations (0,156–10 ng/mL) of recombinant C1qB provided with the ELISA kit.

### Statistical analyses

The analysis was conducted utilizing GraphPad Prism v10.4.2. To ensure adequate statistical analysis, the sample size was determined based on previous experiments [9]. All variables were normally distributed (Shapiro-Wilk test, *P*  > 0.05). Two-tailed Student’s *t* test and, when appropriate, One-way analysis of variance (ANOVA), followed by Tukey’s multiple comparison post hoc test, were performed. All groups that underwent statistical comparisons displayed similar variance. The Pearson’s correlation test was used for correlation analysis. Statistical significance was set at *p* < 0.05. All data were expressed as the mean ± standard deviation (SD), as indicated in the figure legends.

### Ethical approval

All experimental procedures were approved by the Italian Ministry of Health (authorization numbers: 321/2017-PR, protocol number 1F295.34/04-11-2016, 964/2023-PR, protocol number 1F295.182) or by the Ethical Commitee Regione Umbria (Comitato Etico Regione Umbria [CER]), Registro CER: 3933/21, protocol number 20949/21/OV).

## Supplementary information

Supplementary Figures

Additional Table 1

Additional Table 2

Additional Table 3

Addtional Table 4

Additional Table 5

Additional Table 6

## Data Availability

All data generated or analysed during this study are included in this published article [and its supplementary information files]. The sequencing data for this study have been deposited in the GEO (Gene Expression Omnibus) under the accession number GSE317172.
