# Supplementary material for: Transcriptome analysis of the prefrontal cortex identifies inflammatory genes associated with cognitive impairment in a model of multiple sclerosis
Source: Cell Death Discov. 2026 Mar 25;12:177. doi: 10.1038/s41420-026-03051-9 (PMC13039925; doi:10.1038/s41420-026-03051-9)
Supplement: Supplementary file 1 — Supplementary Figures [file 41420_2026_3051_MOESM1_ESM.pdf]

Additional files

File name: **Additional file 1**:

File format: .tif;

Title of data: **Clinical Score and Symptoms (CSS) and forebrain pathology in SJL/J mice during EAE**;

Description of data: (A) Graph shows values of clinical score and symptoms (CCS) in EAE mice evaluated as described in Materials and Methods. Data report the average clinical score for EAE (red) and control (CTRL) (green) mice. All values are given as means ± SD. (B, C) Representative Nissl(B) or Neurotrace- (green) (C) stained coronal sections of EAE-mice brain showing subpial and perivascular infiltrates in the grey- (grey arrows) and white-matter (white arrows), characterized by the presence of CD3+-cells (red-stained) (C). Scale bar: 400 µm in B, 100 µm in C and 50 µm in D-F.

File name: **Additional file 2**:

File format: .tif;

Title of data: **Gene Ontology of differently regulated genes (DEGs) in EAE mice compared with controls**;

Description of data: (A) Biological process and (B) Reactome Pathways 2024 annotations of the DEGs in the PFC of EAE mice compared with controls (CTRLs). (C) Bar graphs showing qPCR analyses performed to validate the upregulation of relevant inflammatory genes in the PFC of EAE mice compared with CTRLs (Student's t Test; $p<0.05$; All values are given as means ± SD).

File name: **Additional file 3**:

File format: .tif;

Title of data: **Heat map representation of the top 100 DEGs in the PFC of EAE-mice**;

Description of data: Heat map representation of the top 100 DEGs in the PFC of control (CTRL, blue) and EAE mice (orange). The color legend indicates the scale of gene expression changes detected (red=upregulated; blue=downregulated). The clustering dendrogram shows the presence of two EAE subgroups, one showing higher differences compared with the CTRL samples (EAE 1, 2 and 7: EAE-H) than the other (EAE 3, 4, 8, 9: EAE-L).

File name: **Additional file 4**:

File format: .tif;

Title of data: **Analysis of disease and PFC transcriptome features of EAE-H and EAE-L mice**;

Description of data: (A) Boxplots showing comparisons of Clinical score and signs (CSS) at the day of sacrifice of EAE-H, EAE-L and control (CTRL) mice. (B) Graph shows values of CCS in EAE-H (red), EAE-L (orange) and CTRL (green) mice evaluated as described in Materials and Methods. Data represent the average clinical score. (C) Boxplots showing comparison of duration of disease (from d.p.i. 0 to the day of sacrifice) of EAE-H and EAE-L mice. No significant difference is evident (Student's t Test; $p<0.05$). (D, E) Heat map representation of the top 100 differently regulated genes in the PFC of CTRL (blue) and EAE-H (D) or EAE-L (E) mice (orange). The color legend indicates the scale of gene expression changes detected (red=upregulated; blue=downregulated). (F, G) Annotations of the DisGeNET database of differently expressed genes (DEGs) in EAE-H (F) and EAE-L (G) subgroups. (H) Bar graphs showing qPCR analyses performed for validation of representative MS-related DEGs in the two EAE subgroups compared with CTRLs, showing their prevalent downregulation in the EAE-H group (One-way ANOVA, Tukey post hoc test, $p<0.05$). All values are given as means ± SD.

File name: **Additional file 5**:

File format: .tif;

Title of data: **Cell-specific expression analysis (CSEA) of downregulated genes shows that high levels of inflammation affect neurons**;

Description of data: (A, B) CSEA (http://doughertytools.wustl.edu/CSEAtool.htm) of downregulated genes in the EAE-L (A) and EAE-H (B) subgroups. (C) Venn diagram of neuron-associated lists of genes in the EAE-H and EAE-L subgroups according to the CSEA tool. (D, E) Annotations of Biological Process (D) and Cellular Component (E) of neuronal genes downregulated in the EAEH subgroup. (F) Annotations of the DisGeNET database of oligodendrocyte-differentially expressed genes in the EAE-H subgroup. (G) Bar graphs showing qPCR analyses performed for validation of representative neuronal genes highlighting their prevalent downregulation in the EAE-H group (One-way ANOVA, Tukey post hoc test, $p<0.05$). All values are given as means ± SD.

File name: **Additional file 6**:

File format: .png;

Title of data: **Features of resident immune cells in EAE-H and EAE-L subgroups**;

Description of data: (A-C) Representative images of Iba1 immunostaining of prefrontal cortex (PFC) coronal sections from control (CTRL) (A), EAE-L (B) and EAE-H mice (C). Increased Iba1^{+} cell density is detectable in both subgroups of EAE animals compared to controls (CTRLs), even more pronounced in EAE-H mice. (D) Bar graphs showing densitometric analysis of Iba1 immunofluorescence in the PFC of CTRLs, EAE-H and EAE-L mice. The F/A ratio defines the mean fluorescence of individual samples (F) normalized to total surface (A). Data are presented as the mean ± SD. (E, F) Representative confocal microscope images of double-stained CD74 (red)/GFAP (green) PFC coronal sections from CTRL (E), EAE-L (F) and EAE-H mice (G). Only some GFAP+ astrocytes show CD74 expression in both subgroups. Scale bar: 100 µm.

File name: **Additional file 7**:

File format: .png;

Title of data: **C1Q is expressed by a subset of microglia/macrophages surrounding inflammatory lesions**;

Description of data: (A,B) Confocal microscope representative images of Iba1 (green) and C1Q (red) double-stained prefrontal cortex (PFC) coronal sections of CTRL (A) and EAE-H (B) mice, counterstained with Dapi (blue). C1Q is exclusively expressed in the PFC of EAE animals. A clear colocalization with Iba1-positive microglia/macrophages located in the inflammatory lesion is evident in (B). The image in B represents an enlargement of the image in the box, encircling an inflammatory lesion in the context of a PFC coronal section immunostained with Iba1(green) and counterstained with Dapi (blue). Scale bar: 100 µm.

File name: **Additional file 8**:

File format: .tif;

Title of data: **Pearson's correlation between expression levels of inflammatory or neuronal genes and cognitive performances of EAE mice**;

Description of data: (A) Pearson's correlation analyses of Clinical score and signs (CSS) of EAE mice on the day of sacrifice (peak of the disease) and the discrimination ratio scored by the same mice in the OIP test. (B) Bar graphs showing qPCR analyses performed for validation of representative genes shown in Fig. 7 C,D. (C) Bar graphs showing qPCR analyses performed for validation of

inflammatory upregulated genes (*Ciita, H2Ab1,H2Eb1, Igpt, C3, C3ar11, Tnf Ccr2, Saa3*) and downregulated neuronal genes (*Pnoc, Bdnf, Nptx2*). Expression levels of selected genes in the PFC of EAE mice are shown associated with graphs representing Pearson's correlation analysis of each gene expression level and discrimination ratio scored by the same mice in the OIP test.

File name: **Additional Table 1**:

File format: .docx;

Title of data: **Primer sequences of genes analyzed by Real Time PCR**;

Description of data: Primer sequences of genes analyzed by Real Time PCR.

File name: **Additional Table 2**:

File format: .xlsx;

Title of data: **List of the differentially expressed genes in the prefrontal cortex of EAE mice**;

Description of data: List of the differentially expressed genes in the prefrontal cortex of EAE mice.

File name: **Additional Table 3**:

File format: .xlsx;

Title of data: **List of the 100 most significantly regulated genes in the prefrontal cortex of EAE mice**;

Description of data: List of the 100 most significantly regulated genes in the prefrontal cortex of EAE mice.

File name: **Additional Table 4**:

File format: .xlsx;

Title of data: **List of the differentially expressed genes in the prefrontal cortex of EAE-H or EAEL mice**;

Description of data: List of the differentially expressed genes in the prefrontal cortex of EAE-H or EAE-L mice.

File name: **Additional Table 5**:

File format: .xlsx;

Title of data: **List of MS-associated genes altered in the PFC of EAE-H and EAE-L mice**;
Description of data: List of MS-associated genes altered in the PFC of EAE-H and EAE-L mice.

File name: **Additional Table 6**:
File format: .docx;
Title of data: **Demographical and clinical characteristics of MS patients analyzed in the study**;
Description of data: Demographical and clinical characteristics of people with MS analyzed in the study.

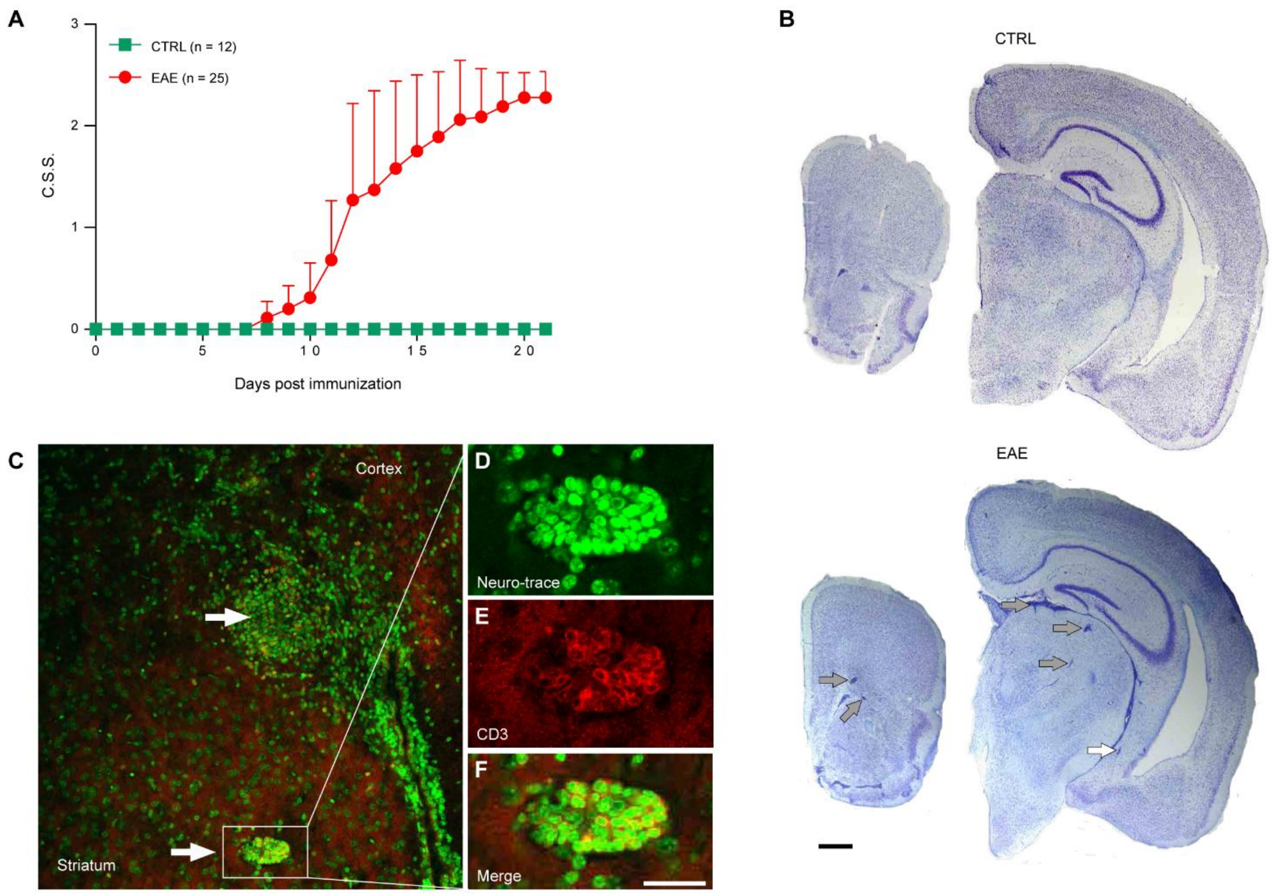

Additional file 1

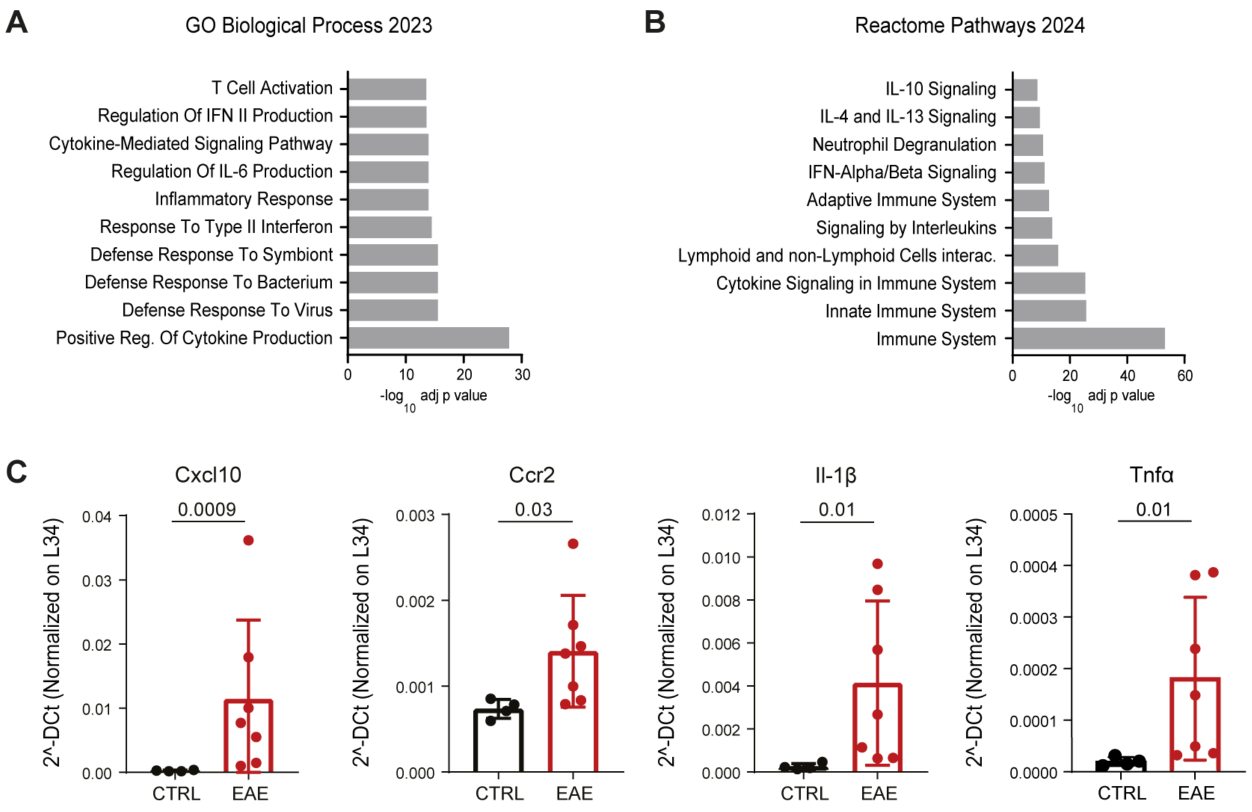

Additional file 2

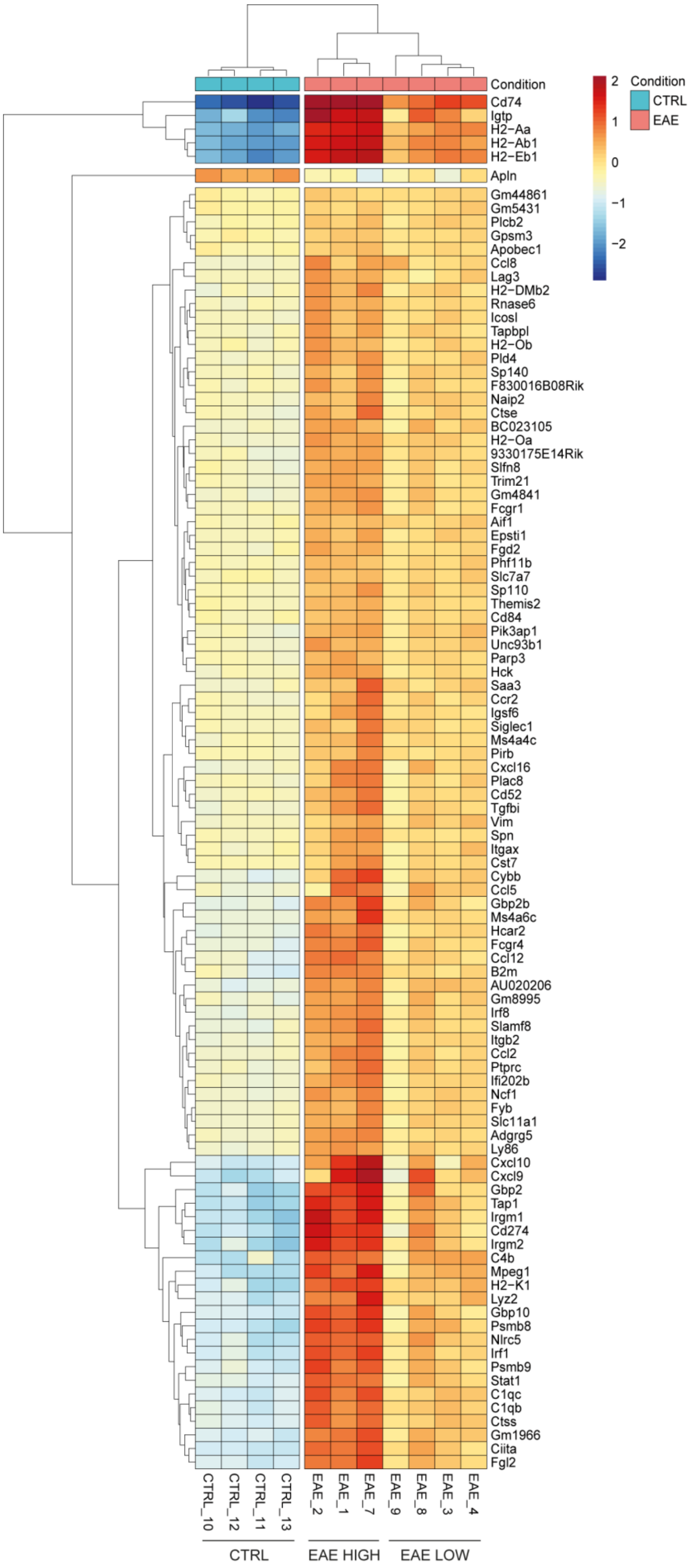

Additional file 3

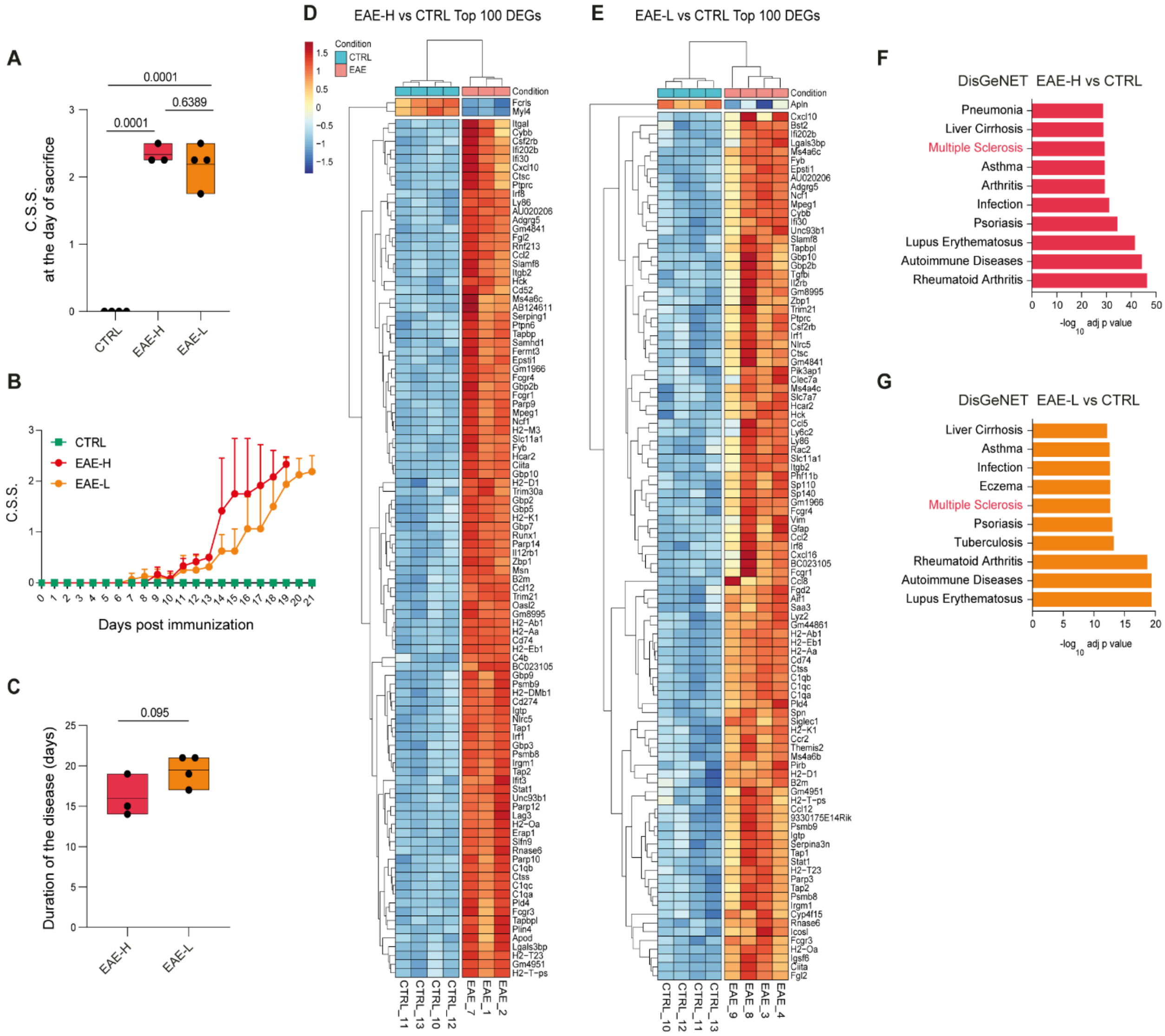

Additional file 4

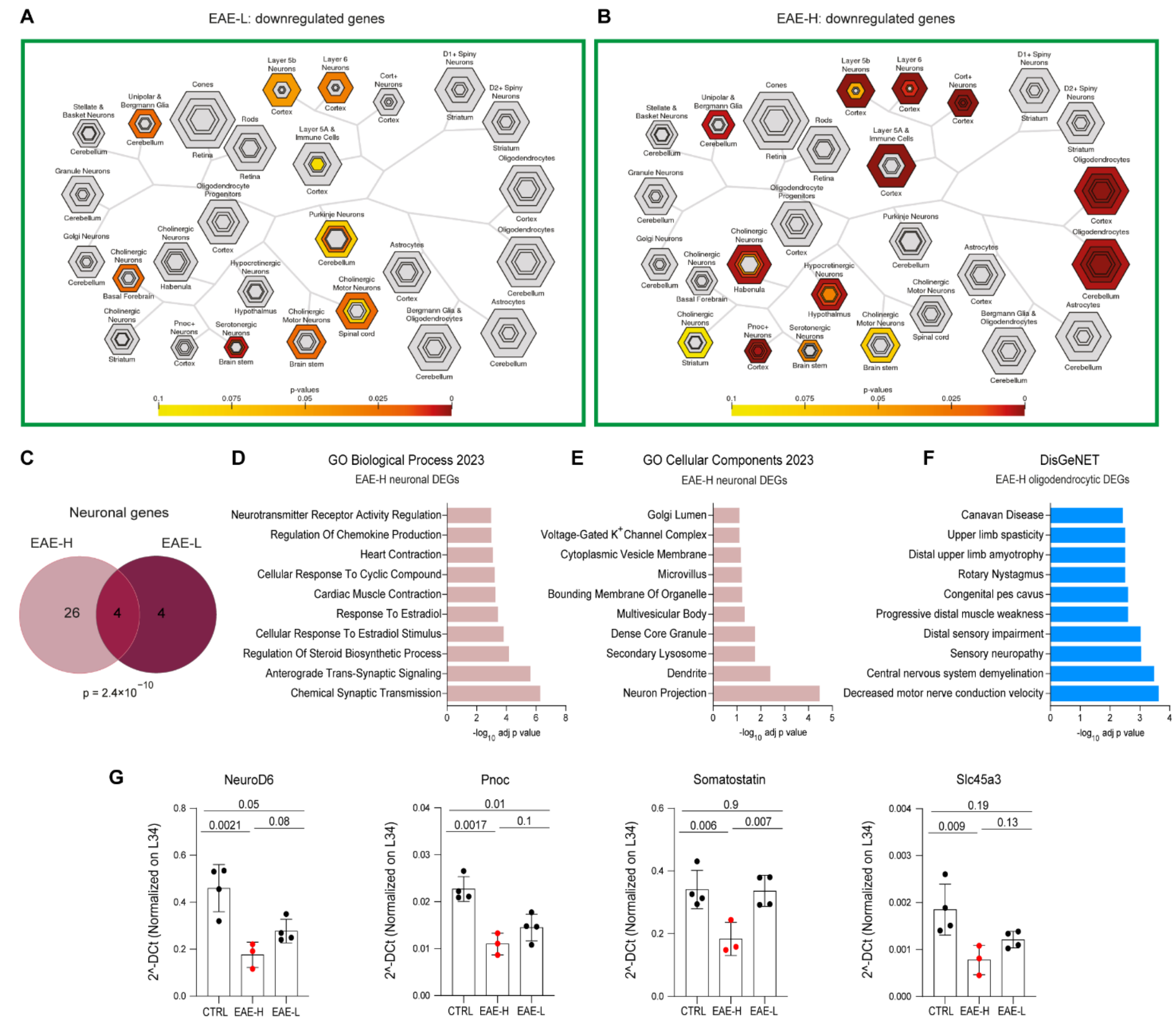

Additional file 5

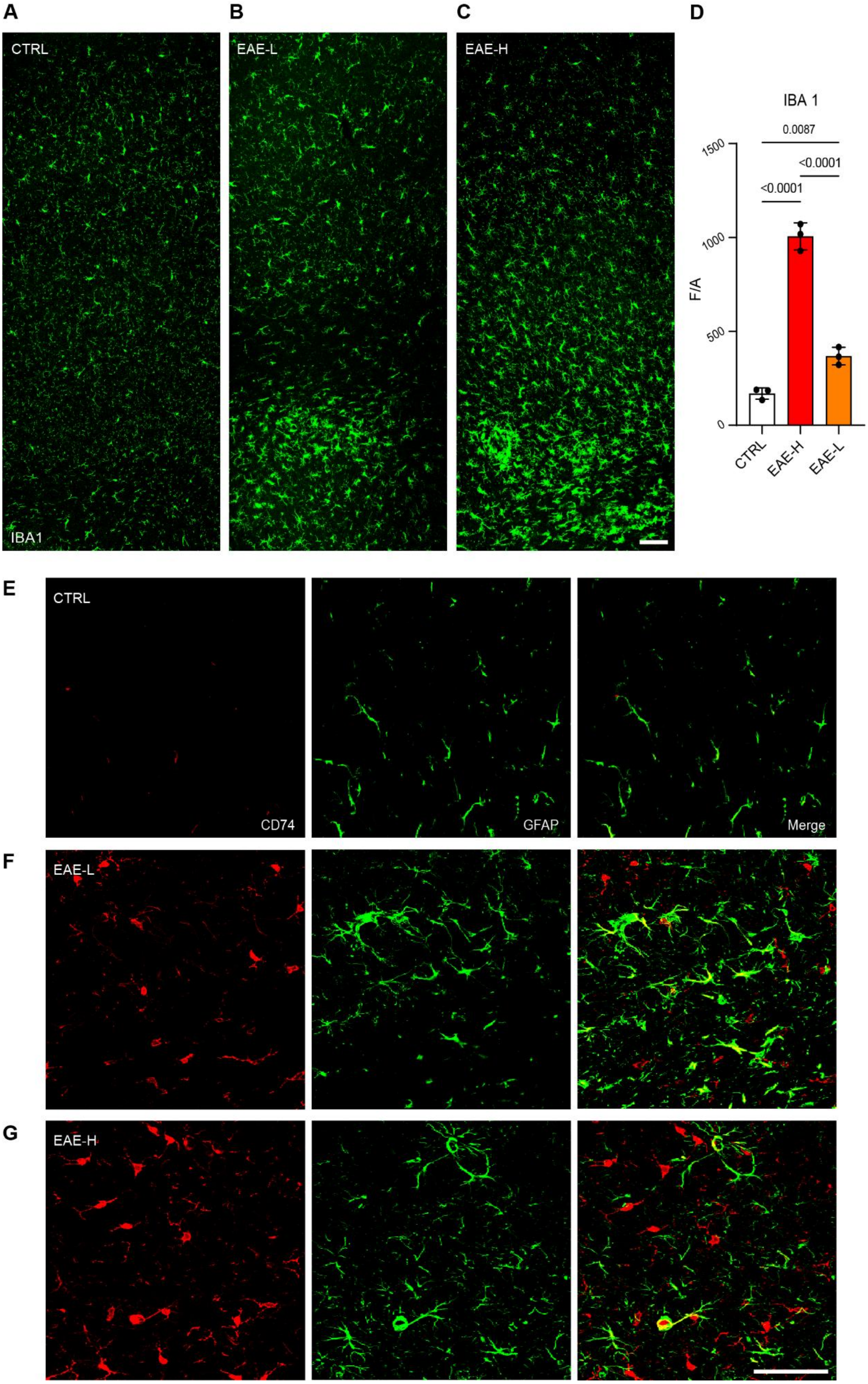

Additional file 6

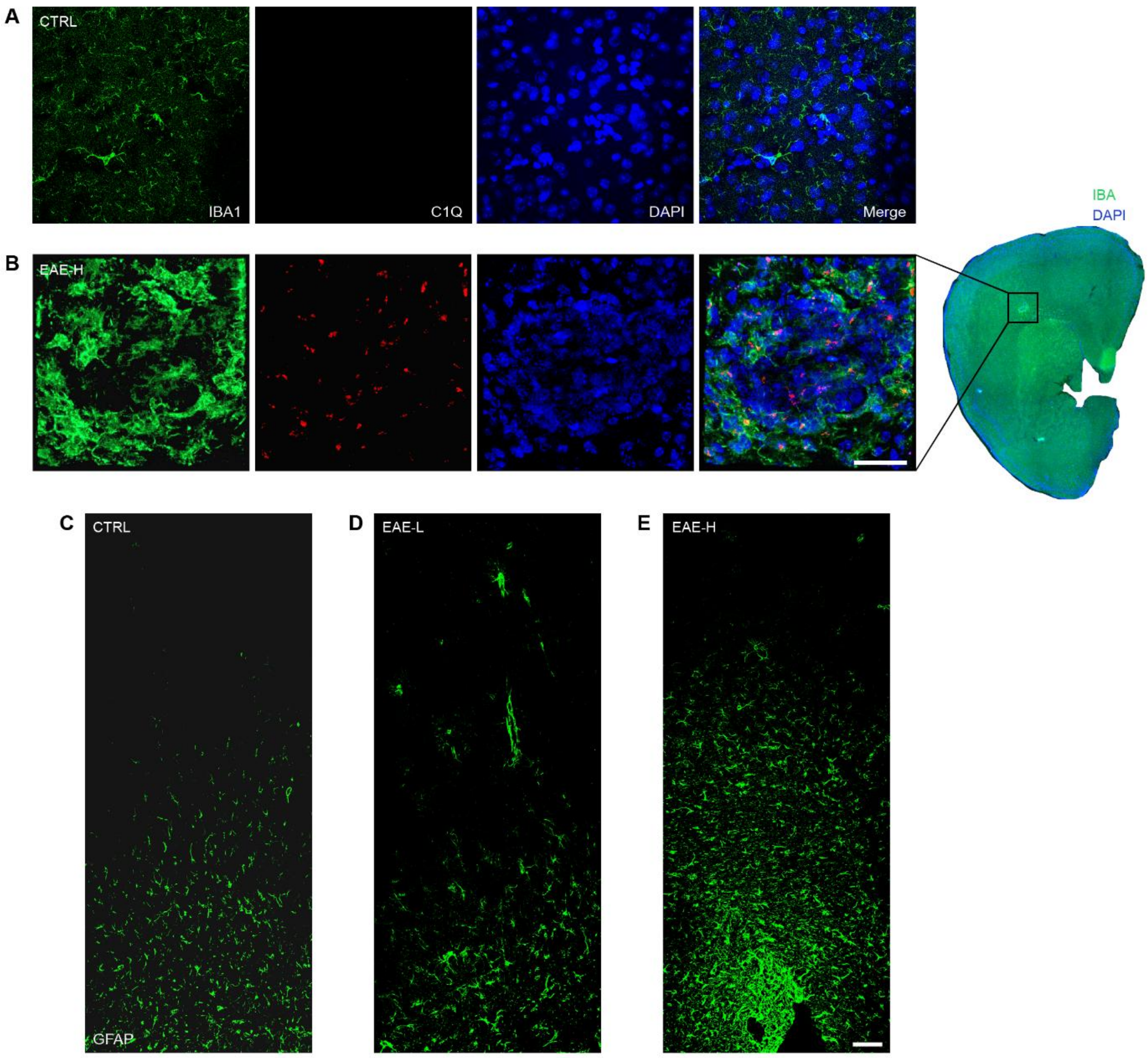

Additional file 7

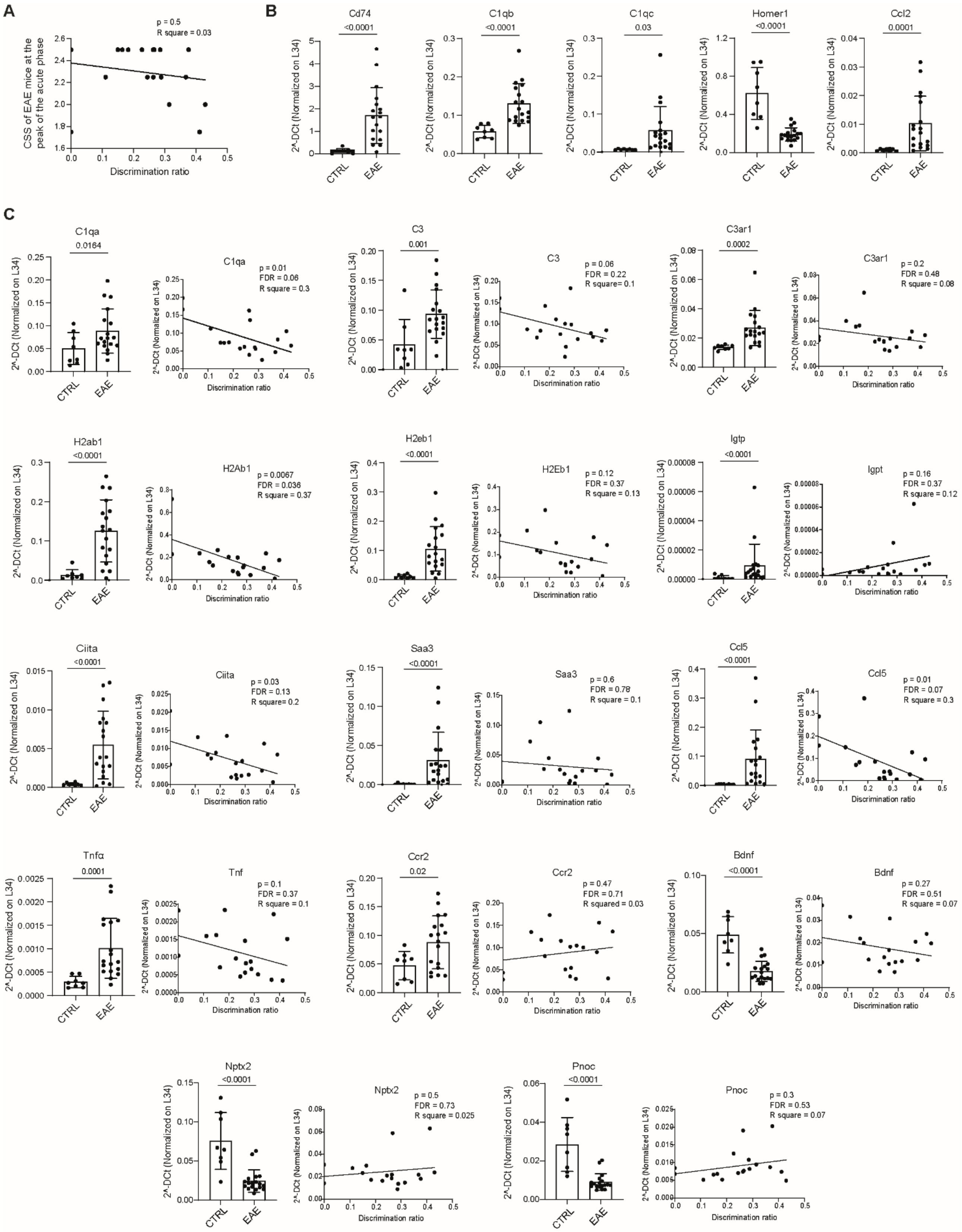

Additional file 8
