# Supplementary material for: Transcriptome analysis of the prefrontal cortex identifies inflammatory genes associated with cognitive impairment in a model of multiple sclerosis
Source: Cell Death Discov. 2026 Mar 25;12:177. doi: 10.1038/s41420-026-03051-9 (PMC13039925; doi:10.1038/s41420-026-03051-9)
Supplement: Supplementary file 2 — Additional Table 1 [file 41420_2026_3051_MOESM2_ESM.docx]

| **Primers' sequences** | **FW** | **RV** |
| --- | --- | --- |
| *Aass* | 5'-CAGCACAGCTTCCAATCGAG-3' | 5'-TGGGGAGAAGTTCTGGCTTTC-3' |
| *Bdnf* | 5'-TACCTGGATGCCGCAAACAT-3' | 5'-GCTGTGACCCACTCGCTAAT-3' |
| *C1qa* | 5'-ATGGAGACCTCTCAGGGATG-3' | 5'-ATACCAGTCCGGATGCCAGC-3' |
| *C1qb* | 5'-AACCAGGCACTCCAGGGATAAA-3' | 5'-GGTCCCCTTTCTCTCCAAACTC-3' |
| *C3* | 5'-GCAGACCTTAGCGACCAAGT-3' | 5'-TTCAGCCATCTGAACCACCG-3' |
| *C3AR1* | 5'-CTCCAGTCACAGCCATTCCTA-3' | 5'-TCTTCAACAGGAAACCCGCTG-3' |
| *Ccl2* | 5’-GGTCCCTGTCATGCTTCTGG-3’ | 5’- GAGTAGCAGCAGGTGAGTGG-3’ |
| *Ccl5* | 5’-GCGGTTCCTTCGAGTGACAA-3’ | 5’-CTGCTGCTTTGCCTACCTCT-3’ |
| *Ccr2* | 5’-CTGCCTCCACTCTACTCCCT-3’ | 5’-GCAGCATAGTGAGCCCAGAA-3’ |
| *Cd74* | 5’-GATGGCTACTCCCTTGCTGA-3’ | 5’-CATCACATGGTCCTGGGTCA-3’ |
| *Cd86* | 5'-GCCACCCACAGGATCAATTA-3' | 5'-TCGGGTGACCTTGCTTAGAC-3' |
| *Ciita* | 5’-CGGCAGAGGAGAAGTTCACC-3’ | 5’-TTGGCTGCATCTTCTGAGGG-3’ |
| *Cxcl10* | 5’-CATTCTCACTGGCCCGTCAT-3’ | 5’-TTTCTGCCTCATCCTGCTGG-3’ |
| *Cyp4f15* | 5'-CTTTGTGATTTCCCTCAACCCC-3' | 5'-GATAGGACCCAGCCATGTCA-3' |
| *Gfap* | 5'-TTTGCAGACCTCACAGACG-3' | 5'-TTGGCGGCGATAGTCGTTA-3' |
| *Gjb1* | 5'-TGCACGTAGCTCACCAACAG-3' | 5'-CGGAACACCACACTGATGACA-3' |
| *Gjc2* | 5'-GTCTTCCGCATTGTGCTGACAG-3' | 5'-GCGTCATAGCAGACGTTGTCAC-3' |
| *H2Ab1* | 5’-GTGTGCAGACACAACTACGAGG-3’ | 5’-CTGTCACTGAGCAGACCAGAGT-3’ |
| *H2Eb1* | 5’-TAAATTCCTTGTGCGGCGGA-3’ | 5’-CTCACAGAGCAGACCAGGAG-3’ |
| *Homer1* | 5'-GTACCCACCAGCAAGCATG-3' | 5'-TCATGTTTGGTGTGATGGTGC-3' |
| *Iba1* | 5'-AAGAGAGGCTGGAGGGGATCAA-3' | 5'-GGCAGATCCTCATCATTGCTGTA-3' |
| *Igtp* | 5’-CCGTGAACAAGTTCCTCAGGCT-3’ | 5’-GAGGTCTTGGTGTTCTCAGCCA-3’ |
| *Il1β* | 5’-TGCCACCTTTTGACAGTGATG-3’ | 5’-ATGTGCTGCTGCGAGATTTG -3’ |
| *Il7R* | 5'-GCAAGGGGTGAAAGCAACTG-3' | 5'-TGGGGAATGGATCGGACTTTG-3' |
| *Irf8* | 5'-GGTATGACCAAGAGGAGCCC-3' | 5'-TCATAGGCGGCATATCCGGT-3' |
| *Kcnv1* | 5’-CAAGAGCTGGAAAATGTGGGACG-3’ | 5’-CATAGCACTGAGTGATTGTCATCC-3’ |
| *L34* | 5’-GGTGCTCAGAGGCACTCAGGATG-3’ | 5’-GTGCTTTCCCAACCTTCTTGGTGT-3’ |
| *NeuroD6* | 5’-ATGCGACACTCAGCCTGAAA-3’ | 5’-CTGGGATTCGGGCATTACGA-3’ |
| *Nptx2* | 5’-GGCAAGCCAACGAGATTGTGCT-3’ | 5’-TGCCATCACTGACAAACAGCGG-3’ |
| *Pnoc* | 5’-TGGTGCATACAGTCCAGAGA-3’ | 5’-TTTGTGACGTTCTGCTGCTC-3’ |
| *Saa3* | 5’-TGATCCTGGGAGTTGACAGC-3’ | 5’-CCCCCGAGCATGGAAGTATT |
| *Slc45a3* | 5'-AGGCCCGGAGACACTATGAT-3' | 5'-GGCCAGATAGACTGACCGTG-3' |
| *Sst* | 5’-AGAGAATGATGCCCTGGAGC-3’ | 5’-CATTGCTGGGTTCGAGTTGG-3’ |
| *Tnfα* | 5'-CGAGTGACAAGCCTGTAGCCC-3' | 5'-GTCTTTGAGATCCATGCCGTTG-3' |
