# Supplementary material for: Transcriptome analysis of the prefrontal cortex identifies inflammatory genes associated with cognitive impairment in a model of multiple sclerosis
Source: Cell Death Discov. 2026 Mar 25;12:177. doi: 10.1038/s41420-026-03051-9 (PMC13039925; doi:10.1038/s41420-026-03051-9)
Supplement: Supplementary file 7 — Additional Table 6 [file 41420_2026_3051_MOESM7_ESM.docx]

|  | **All (n: 40)** | **CI- (n: 20)** | **CI+ (n: 20)** | **p-value*** |
| --- | --- | --- | --- | --- |
| **Demographical characteristics** | | | | |
| Age (years) | 40.1 ± 13.8 | 35.5 ± 6.8 | 40.1 ± 13.8 | n.s. |
| Sex (F) – N (%) | 26 (65) | 13 (65) | 13 (65) | n.s. |
| **Clinical characteristics** | | | | |
| Disease duration (months) | 6.9 ± 10.5 | 6.1 ± 7.5 | 6.9 ± 10.5 | n.s. |
| EDSS | 1.8 ± 0.9 | 1.7 ± 0.8 | 1.9 ± 1 | n.s. |
| CSF IgG OCB+ - (% of patients) | 36 (90) | 18 (90) | 18 (90) | n.s. |
| Brain Gd+ lesions - (% of patients) | 21 (52.5) | 10 (50) | 11 (55) | n.s. |

*Comparison between CI- and CI+ pwMS.

**Legend.** CI-: pwMS without cognitive impairment. CI+: pwMS with cognitive impairment. CSF: cerebrospinal fluid. EDSS: Expanded Disability Status Scale. Gd+: gadolinium enhancing. IgG: immunoglobulin G. OCB: oligoclonal bands.
